# Adsorption Sites on Pd Nanoparticles Unraveled by Machine-Learning Potential with Adaptive Sampling

**DOI:** 10.3390/molecules27020357

**Published:** 2022-01-06

**Authors:** Andrei Tereshchenko, Danil Pashkov, Alexander Guda, Sergey Guda, Yury Rusalev, Alexander Soldatov

**Affiliations:** 1 The Smart Materials Research Institute, Southern Federal University, 344090 Rostov-on-Don, Russia; pashkov@sfedu.ru (D.P.); guda@sfedu.ru (A.G.); gudasergey@gmail.com (S.G.); yuri.rusalev@gmail.com (Y.R.); soldatov@sfedu.ru (A.S.); 2 Vorovich Institute of Mathematics, Mechanics, and Computer Sciences, Southern Federal University, 344058 Rostov-on-Don, Russia

**Keywords:** palladium nanoparticles, probing molecules, adsorption energy, machine learning, radial distribution function, adaptive sampling

## Abstract

Catalytic properties of noble-metal nanoparticles (NPs) are largely determined by their surface morphology. The latter is probed by surface-sensitive spectroscopic techniques in different spectra regions. A fast and precise computational approach enabling the prediction of surface–adsorbate interaction would help the reliable description and interpretation of experimental data. In this work, we applied Machine Learning (ML) algorithms for the task of adsorption-energy approximation for CO on Pd nanoclusters. Due to a high dependency of binding energy from the nature of the adsorbing site and its local coordination, we tested several structural descriptors for the ML algorithm, including mean Pd–C distances, coordination numbers (CN) and generalized coordination numbers (GCN), radial distribution functions (RDF), and angular distribution functions (ADF). To avoid overtraining and to probe the most relevant positions above the metal surface, we utilized the adaptive sampling methodology for guiding the ab initio Density Functional Theory (DFT) calculations. The support vector machines (SVM) and Extra Trees algorithms provided the best approximation quality and mean absolute error in energy prediction up to 0.12 eV. Based on the developed potential, we constructed an energy-surface 3D map for the whole Pd_55_ nanocluster and extended it to new geometries, Pd_79_, and Pd_85_, not implemented in the training sample. The methodology can be easily extended to adsorption energies onto mono- and bimetallic NPs at an affordable computational cost and accuracy.

## 1. Introduction

Palladium nanoparticles (NPs) are famous catalysts for various reactions [1]. Their catalytic properties significantly depend on size and shape [2,3,4], which could be estimated by the molecular adsorption techniques. In particular, CO pulse chemisorption enables determination of particle dispersion by their CO uptake [5,6]. Fourier-transform infrared (FTIR) spectroscopy of adsorbed CO probes various adsorption sites, such as extended surfaces Pd (111) and (100) and different defects (corners, edges) [7,8]. This is possible due to the strong dependency of vibration frequency from the local environment of adsorbing site, leading to different binding (or adsorption) energies [9,10,11]. Conventionally, theoretical frequencies and binding energies of adsorbed molecules are obtained from Density Functional Theory (DFT) calculations [12,13,14,15,16]. However, even for single-molecule adsorption, such DFT computations can require a lot of computational resources to describe the whole surface of the NPs.

Machine learning (ML) has already demonstrated a high potential in material science. In particular, it was used for predicting and analyzing X-ray absorption spectra [17], where it succeeded in predicting coordination numbers (CN) and radial distribution functions (RDF) [18,19] of monometallic NPs and reconstruction of the structure of bimetallic NPs with atomic resolution [20]. Lansford and Vlahos [21] applied polynomial regression using neural network ensembles to extract information about the microstructure of NPs from FTIR spectra. The authors collected a training set of ab initio-calculated FTIR spectra of CO and NO molecules adsorbed on platinum nanocatalysts, and selected the frequencies and intensities of CO and Pt–C and generalized coordination numbers (GCN) [22] as microstructure descriptors to obtain detailed information on adsorption sites. As a result, they successfully predicted the centers where adsorption occurs for the experimentally measured spectra and the distribution functions of GCN, thereby establishing surface coordination.

Gasper et al. [23] applied the gradient-boosting regression algorithm to predict the adsorption energies in different regions of Pt NPs of various sizes (0.2–1.5 nm). For this purpose, they used a combination of both structural (GCN of adsorption centers, cluster size, bond lengths) and electronic (the position of the center of the d-band, energies of completely frozen structures introduced by the authors) descriptors. This approach allowed them to determine the binding energies of adsorbates with an error comparable with the results of DFT calculations.

Recently, Praveen and Comas-Vives [24] designed a highly accurate ML algorithm, able to predict the adsorption energies of several adsorbates binding either via C, N, O, or H on the surface sites of (100), (111), and (211) facets of transition-metals simultaneously. They combined electronic and structural descriptors (such as CN, GCN, and others) of free adsorbates on clean metal surfaces (10–13 features in total) and applied extragradient boost regression in combination with a tree booster to obtain the best performance.

However, all the abovementioned studies rely to one degree or another on some ab initio (or Monte-Carlo) calculations for the preparation of training sets. Among the classical approaches to the selection of training set points are grid sampling, random sampling, and Improved Latin Hypercube sampling (HIS) [25]. However, the use of homogeneous methods for sampling may underestimate the importance of small regions on the surface near the adsorption sites. With such important for catalysis points, the method would make mistakes but the overall statistical precision of the approach would still be high due to the low fraction of such points. Enlarging the training set for denser sampling requires a lot of additional time and computational efforts.

In this work, we applied adaptive sampling (also called active learning or response-adaptive designs) for effective training-set generation. This approach has already demonstrated high performance in molecular dynamics and simulations [26,27] and material design [28]. Gastegger et al. [29] used adaptive sampling for the preparation of training sets by ab initio molecular-dynamics simulations for infrared spectra prediction, and it enabled acceleration of simulations by several orders of magnitude and extending the size of treated systems. The method is based on stepwise addition of the new points in the training set when cross-validation quality suggests the areas for denser sampling. We discuss the efficiency of different ML algorithms such as Ridge regression, Decision tree, support vector machines (SVM), Least absolute shrinkage and selection operator (Lasso), and several ensemble methods (Decision tree, AdaBoost, XGBoost, Random forest, Gradient boosting). RDF and angular distribution functions (ADF) were used as descriptors and compared with conventional descriptors such as CN and GCN along with the mean distance and Coulomb matrix. The developed approach enabled prediction of binding energy with high precision and construction of an energy-surface map for Pd NPs of different sizes.

## 2. Methods

### 2.1. DFT Computation Details

All DFT calculations were carried out using the Vienna Ab initio simulation package (VASP) code [30,31] and the projector augmented wave (PAW) method [32] for periodic structures.

An octahedral Pd_55_ nanocluster was placed in the center of the cell with a size of 30 ×30 × 30 Å^3^. The calculations were performed using 1 k-point in a direct space with an energy cutoff of 400 eV inside a cubic supercell. Geometry optimization of the atomic positions was performed with a criterium of energy convergency of 10^−6^ eV and force convergency of 10^−2^ eV/Å.

Obtained total energies were recalculated to binding energies using the equation (Equation (1)):

(1) $$E_{bind}=E_{NP+\mathrm{CO}}-\left(E_{NP}+E_{CO}\right)$$

where *E*_*bind*_ is binding energy, *E*_*NP*+CO_ is the total energy of Pd_55_ with adsorbed CO, *E*_*NP*_ is the energy of Pd_55_ without adsorbate, and *E*_*CO*_ is the energy of the free CO molecule.

For benchmarking, the DFT calculations were performed for four different sites at (100) and (111) facets and two sites at defects of Pd_55_:

- the central atom of Pd(100)–(100)—top
- between two atoms of Pd(100)–(100)—bridge
- between two atoms of Pd(111)–(111)—bridge
- between three atoms of Pd(111)–(111)—hollow (three-fold)
- between two atoms of the edge between Pd(100) and Pd(111)—edge bridge
- on the single atom of the corner—vertex top

Single CO molecules (one for each calculation) were placed on these sites by carbon end, and potential energy scans were performed upon moving CO from 0.1 to 5 Å from these sites (Figure S1 of Section 1, Supplementary Materials) with a step of 0.1 Å. The positions of the carbon and Pd atoms were fixed, while positions of oxygen atoms were optimized for each step before energy calculation. The initial C–O distance was set to 1.128 Å.

When calculating the binding energy by the DFT approach, computational errors are mostly associated with pseudopotential and the form of the exchange–correlation functional.

The DFT-GGA calculations of CO adsorption on transition metals [33] tend to favor higher coordination sites, leading to a site preference error mainly due to the core overlap between metal and CO. Mason et al. [34,35] estimated this error as 0.38 eV (~30%) for the PBE functional and developed an approximation approach enabling a decrease in average error to 0.16 eV (~13%). Revised versions of semi-local functionals partially address this problem. According to results of CO adsorption modeling on fcc sites of Pd(111) reported by Hammer et al. [36] and performed under the revised Perdew-Burke-Ernzerhof (rPBE) exchange-correlation functional [37,38], the overbinding was estimated as 0.1–0.2 eV. This functional has been specifically designed to improve upon over-binding issues, particularly tailored to the chemisorption of CO [39]. A GGA rPBE exchange–correlation functional was also applied in this work.

### 2.2. Descriptors of Structure

RDF, or pair correlation function $g\left(r\right)$ of a system of particles (atoms), is the probability of finding a particle at a distance r from another tagged particle. It describes how density varies as a function of distance from a reference atom, and its calculation is one of the most common methods of describing the structure of a system. In this work, we applied RDF of Pd atoms relative to the carbon atom that belongs to the adsorbing molecule of carbon monoxide, as a descriptor of structure for ML. All RDF were calculated using vasppy [40] and pymatgen [41,42] Python modules (see Section 2, Supplementary Materials, for detailed information).

The following structural parameters were evaluated for each structure apart from RDF:

- mean distance from carbon to the nearest Pd atoms (<d_Pd–C_>)
- CN of the carbon atom of CO molecule;
- GCN of adsorbing site;
- ADF for Pd–C–Pd and Pd–C–O combinations.

The problem of energy approximation is a typical regression problem. Each object of the training set was represented by values of RDF determined in the range from 0 to 7 Å with a step of 0.01 Å, and values of ADF in the phi range from 0 to 180 ° with a step of 1°.

### 2.3. Training- and Test-Set Preparation

Spherical coordinates were applied to describe the positions of CO molecules around Pd NP. The parameter space was represented by θ and φ angles, and the distance R from the Pd NP surface to the carbon atom R_Pd–C_. Upon R, θ, and φ variations, we kept the minimal Pd–C distance R_min_ equal to 1.62 Å and maximal distance R_max_ = 3.02 Å. This region represents the valley of a minimum of the attractive potential. Details of points generations for training-set preparation are shown in Section 2, Supplementary Materials. The training set was prepared following the procedure of adaptive sampling described in Section 2.4.

A set consisting of 500 unique structures with the random location of CO in the region of parameter variation (θ in [0, π], φ in [0, 2π], and R_Pd-C_ in [0, 1.4 Å]) was generated. We will refer to it as a Random Sampling Test Set (RSTS).

### 2.4. Adaptive Sampling

The aim of applying ML methods is to find the model that enables one to approximate a hidden dependency in data. Let $\hat{y}$ be an unknown binding-energy dependence on RDF which is defined on D⊂ℝ, and $x_{i}$—a set of spherical coordinates $\left\{r_{i\;},\theta_{i},\;\phi_{i}\right\}$. Then, $X^{l}={\left\{\left(x_{i},y_{i}\right)\right\}}_{i=1}^{l}$ where $y_{i}=\hat{y}\left(x_{i}\right)$ will be the training dataset of size *l.* This dataset is used to build an approximation model a:D→ℝ via ML method $\mu :a=\mu \left(X^{l}\right)$.

**Scheme 1:**
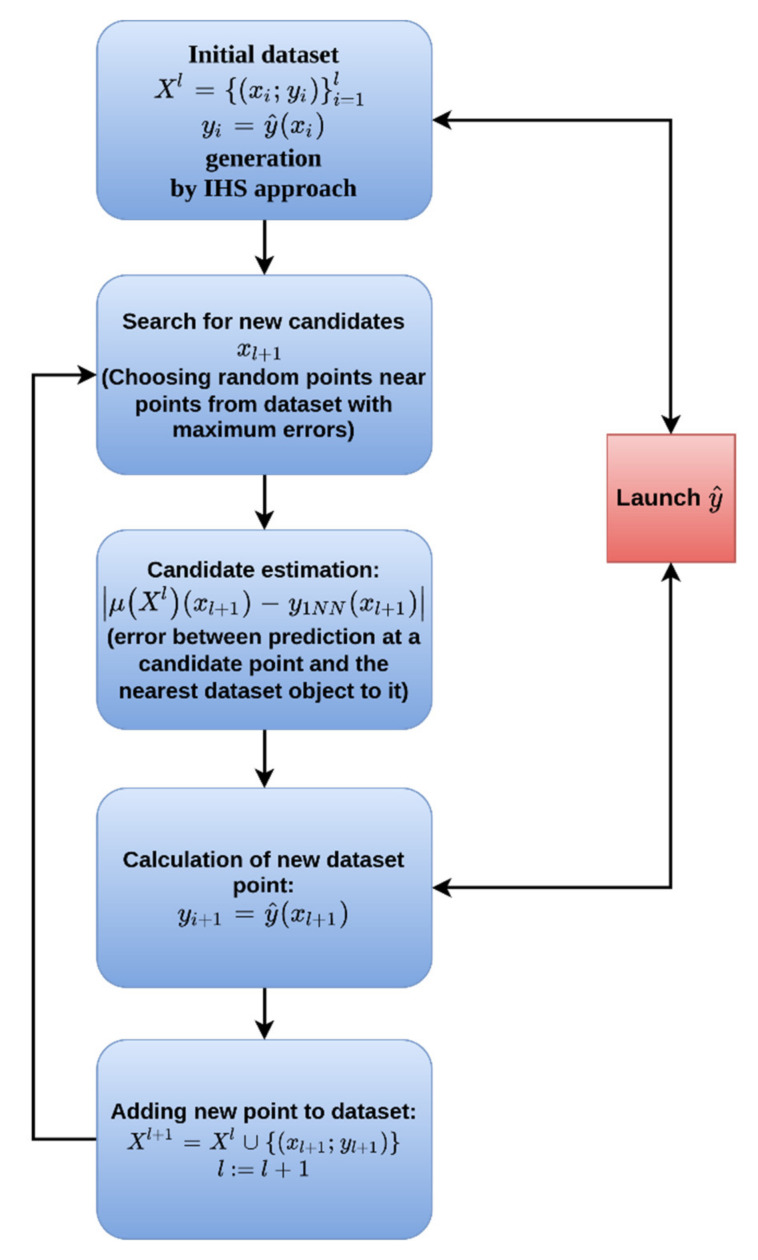
Algorithm of adaptive sampling.

Since the DFT calculations of the binding energies are time-consuming, it becomes necessary to use effective approaches for the training-set preparation, and not just calculate all possible or some random points around metal NP. In this work, we applied an adaptive sampling approach to generate a training set as small as possible, which would be enough to train ML methods for binding-energy prediction with satisfactory quality. The algorithm of its work is presented in Scheme 1.

At the beginning of its work, the adaptive sampling method generates homogeneously some points using IHS. This training set $X^{l}$ is used to train an ML method $\mu \left(X^{l}\right)$, which is the first approximation of $\hat{y}$ dependence. This model predicts the binding energy and the quality of model predictions depending on the training dataset. In this study, the Extra Trees ML method was used as a μ function during the training-dataset-generation process.

To improve the generalizing ability of the model, it is necessary to expand the training dataset. It is necessary to choose the next nodal point $x_{l+1}$ so that the new approximation constructed from the sample $X^{l+1}=X^{l}\cup \left\{x_{l+1};y_{l+1}\right\}$ approximates the target dependence $\hat{y}$ in the best way (Equation (2)):

(2) $$||\mu \left(X^{l+1}\right)-{\hat{y}||}_{L_{p}\left(D\right)}\to \begin{array}{c} min\\ x_{l+1} \end{array}$$

To find a good point, we should try to keep the balance between local exploitation and global exploration. The global exploration term votes for placing new sampling points inside unvisited regions and the local exploitation term votes for a thorough investigation of the most interesting regions. The key to solving this problem is the following formula (Equation (3)):

(3) $$\overset{\;}{\underset{S\left(x_{l+1}\right)}{\int }}{\left|\mu \left(X^{l}\right)-\hat{y}\right|}^{P}\to \begin{array}{c} max\\ x_{l+1} \end{array},$$

where $S\left(x_{l+1}\right)$ is the vicinity containing $x_{l+1}$.

Candidates for the next point are generated in the vicinity of each of the maxima of the approximation error in the range of distances $\left[0.5d_{1NN};2.5d_{1NN}\right]$, where $d_{1NN}$ is the distance to the nearest neighbor of the point with the maximum approximation error.

The next important factor in optimization is the approximation error estimation $\left|\mu \left(X^{l}\right)\left(x\right)-\hat{y}\left(x\right)\right|$. A more universal, widespread approach is based on cross-validation. To save time, the calculation of the integral in Equation (3) was replaced by a point estimate of the approximation error divided by the density of points in the vicinity of $x_{l+1}$. To estimate the error from Equation (2), the following expression has been used (Equation (4)):

(4) $$\left|\mu \left(X^{l}\right)\left(x_{l+1}\right)-y_{1NN}\left(x_{l+1}\right)\right|$$

where $y_{1NN}\left(x_{l+1}\right)$ is the point of the training sample closest to the $x_{l+1}$ candidate.

After determining the best candidate for which an approximation error is the highest, it is included in the training dataset. According to this algorithm, an adaptive sample is formed sequentially.

We will refer to the set generated using this approach and consisting of 548 unique data points as the Adaptive Sampling Training Set (ASTS). Examples of these structures can be found in Section 3, Supplementary Materials.

### 2.5. Assessment of Prediction Quality

Nine different ML methods such as a linear methods of Ridge regression and Lasso [43], Decision tree [44], SVM [45,46], and five different ensemble methods: Gradient boosting [47], Extra trees [48], XGBoost [49], AdaBoost [50], and Random forest [51], were trained on ASTS for the task of binding-energy prediction. All ML algorithms were implemented using the Scikit-learn package for Python [52,53]. RidgeCV and LassoCV modifications were used to protect these linear methods from overtraining.

The effectiveness of the ML methods trained on the ASTS was tested on RSTS data (see Section 2.3). Thus, a test error was determined on external data.

The quality of predictions was assessed using three quality metrics (Equations (5)–(7)): R^2^-score, mean absolute error (MAE), and mean square error (MSE):

(5) $$R^{2}=1-\frac{\sum_{i=1}^{N}||E_{i}-{\hat{E_{i}}||}^{2}}{\sum_{i=1}^{N}||E_{i}-{\langle E\rangle ||}^{2}}$$

(6) $$MAE\left(E,\hat{E}\right)=\frac{1}{N}\overset{N}{\underset{i=1}{\sum }}\left|E_{i}-\hat{E_{i}}\right|$$

(7) $$MSE\left(E,\hat{E}\right)=\frac{1}{N}\overset{N}{\underset{i=1}{\sum }}{\left(E_{i}-\hat{E_{i}}\right)}^{2}$$

where $E_{i}$ are the ab initio DFT energy values, $\hat{E_{i}}$—are the values predicted by ML binding energies, and 〈E〉 is the average energy value calculated as (Equation (8)):

(8) $$\langle E\rangle =\frac{1}{N}\overset{N}{\underset{i=1}{\sum }}E_{i\;}$$

## 3. Results and Discussion

### 3.1. Prediction Binding Energy Using ASTS

**Figure 1:**
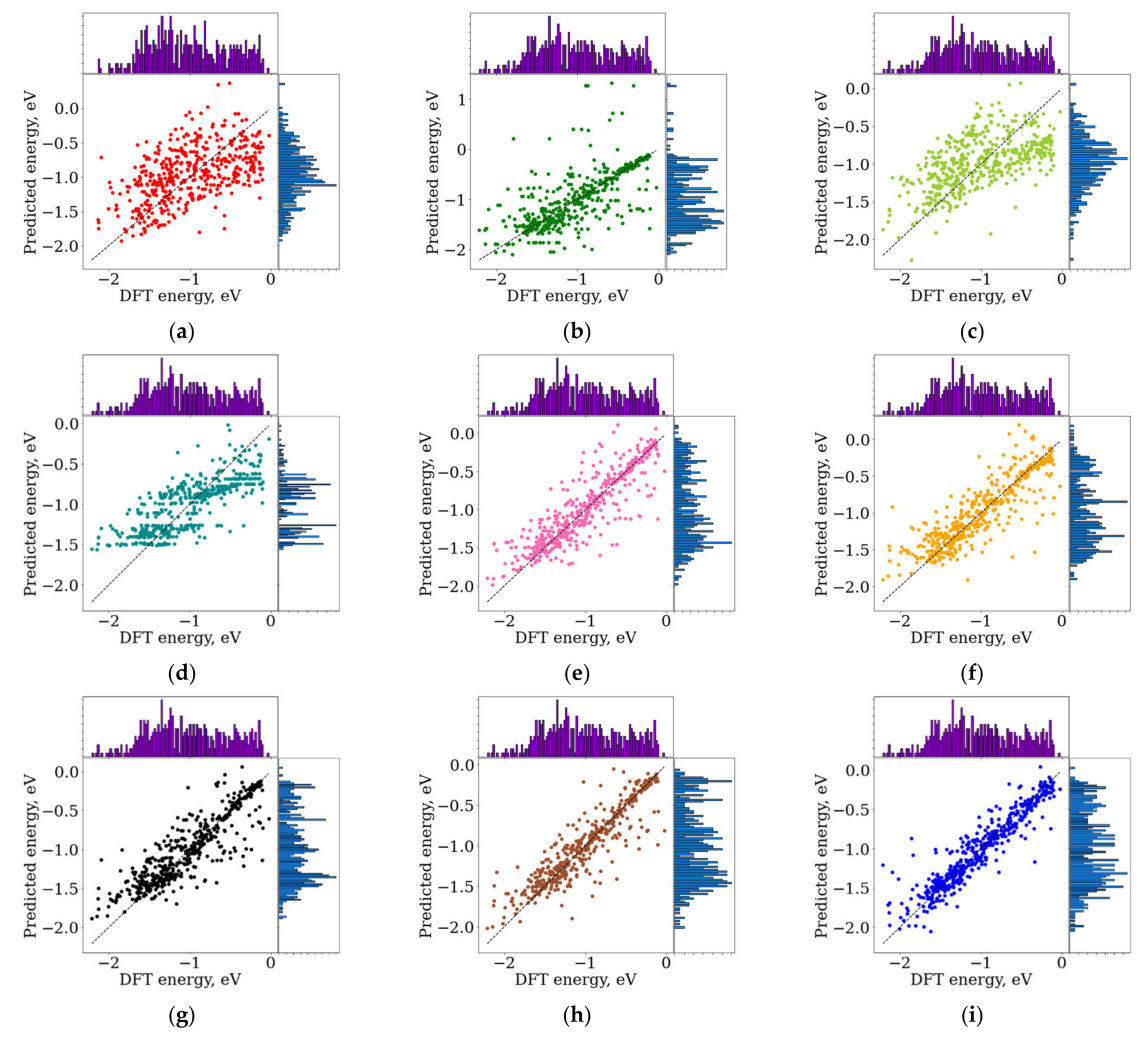
Machine learning (ML)-predicted vs. Density Functional Theory (DFT)-calculated binding energy in eV for different ML algorithms: (**a**) Ridge regression; (**b**) Decision tree; (**c**) Least absolute shrinkage and selection operator (Lasso); (**d**) AdaBoost; (**e**) XGBoost; (**f**) Gradient boosting; (**g**) Random forest; (**h**) Extra trees; (**i**) support vector machines (SVM). The entire interval of radial distribution functions (RDF) 0–7 Å from the carbon atom of CO was used as a descriptor. Histograms on the opposite axis represent distributions of respective values of energy.

Results of binding-energy predictions by different ML methods are shown in Figure 1, depicting ML predicted energy vs. the binding energy calculated at the DFT level. Respective quality metrics are summarized in Table 1. The entire interval of RDF (radius 0–7 Å from carbon atom in CO) was used as a descriptor of structure (Figure S2, Supplementary Materials). This choice of a descriptor is explained further, in Section 3.2.

The best quality was achieved by the SVM algorithm, for which MAE was close to 0.15 eV. It is comparable or even less than an underlying error of DFT calculations of binding energies [23]. The ensemble methods were somewhat less efficient for energy prediction. The worst quality of prediction was demonstrated by the Ridge regression and Lasso methods, which may be due to the complex dependence of the binding energy on the RDF when the linear model is insufficient.

### 3.2. Comparison between Structural Descriptors

**Figure 2:**
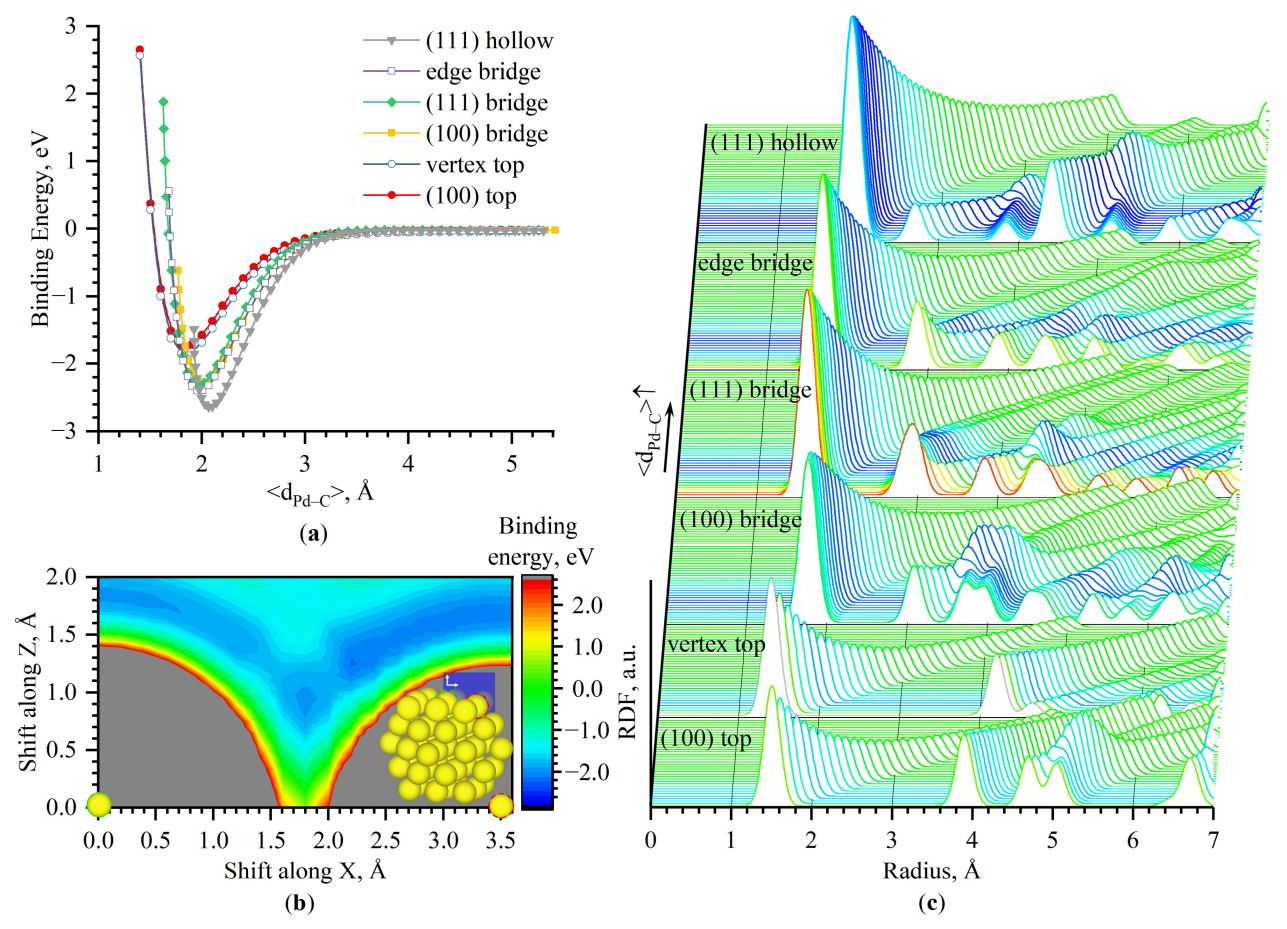
DFT potential energy scans upon moving CO molecules (**a**) from adsorption sites on the surface of Pd_55_ nanocluster; (**b**) along the plane perpendicular to Pd(100) (nearest Pd atoms are marked by red and green dashes). (**c**) Series of smeared RDF upon CO moving away from the respective adsorbing site, colored according to binding energies. The color scale is the same for parts (**b**,**c**).

The energy of adsorption is influenced mostly by the local coordination of adsorbed molecule (adsorbing site) and the distance to this site, but it also depends on a neighborhood of Pd atoms forming the adsorbing site. To illustrate these effects, potential energy scans (Figure 2a,b) were performed for different adsorption sites of Pd_55_ nanoclusters (see structures in Figure S1, Supplementary Materials) both upon CO molecules moving away from these sites and moving along the plane perpendicular to Pd (100). The binding energy decreases for molecules adsorbed on hollow > bridge > top positions and varies by almost 1 eV for carbonyls formed on different sites. Furthermore, adsorption on sites with a similar local environment has different binding energies: in particular, binding on the top position of corner defects of Pd NP will be slightly stronger than on regular Pd(100) surface, and CO adsorbed on different bridged sites will have different adsorption energy, even if this difference is an order of magnitude smaller.

The mean distances <d_Pd–C_> where binding energy reaches minima increase from 1.8 (top) to 1.96 (bridge) and 2.06 Å (for hollow sites). However, <d_Pd–C_> differs negligibly for sites with a similar local environment, e.g., bridged sites on different facets or edges. Energy scans performed along the plane perpendicular to the Pd(100) facet of Pd_55_ (see Figure 2b) demonstrated that binding energy changes significantly for scans in directions perpendicular to the surface (Z in Figure 2b), but there are areas with close energy values upon moving simultaneously in Z and X directions (parallel to the Pd(100) surface).

Thus, distinguishing sites with similar local environments and areas with close but different energies is essential for precise binding-energy prediction but cannot be done using only <d_Pd–C_> as a descriptor.

As is clear from Figure 2c, the shape of RDF for different distances between CO and the adsorbing sites of Pd_55_ are sensitive both to energies and mean Pd–C distances (<d_Pd–C_>) distances. Therefore, RDF is expected to be an adequate descriptor for energy approximation.

**Figure 3:**
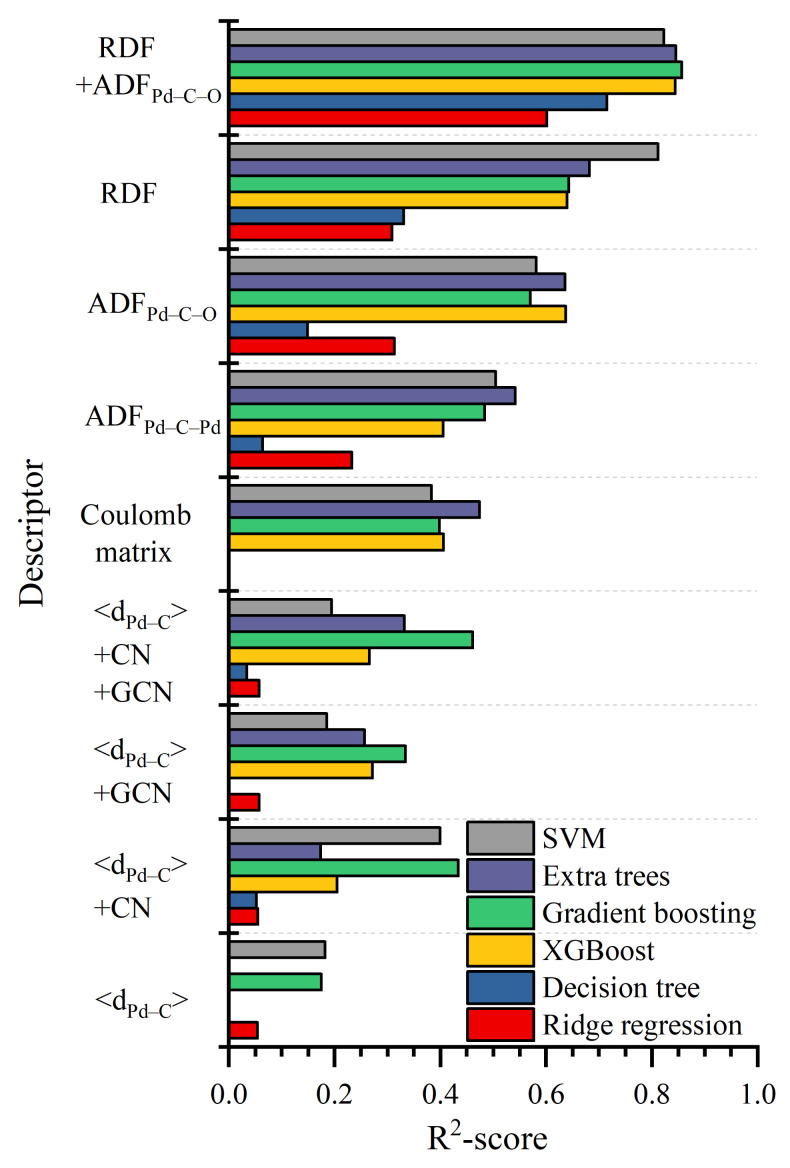
Comparison of the quality of prediction of binding energy when using different descriptors/ML methods.

We compared results of binding-energy predictions made by using RDF as a descriptor and other conventional structural descriptors such as Coulomb matrix and <d_Pd–C_> combined with CN of carbon and/or GCN of adsorbing site. Furthermore, we tested the efficiency of ADF used as a descriptor (Figure S3, Supplementary Materials). Results of their comparison are summarized in Figure 3 and Table S3, Supplementary Materials (results obtained by Lasso and other ML methods, are shown in Figure S5, Supplementary Materials).

We found that <d_Pd–C_> were ineffective descriptors. CN and GCN cannot be directly used as descriptors themselves because they are discrete and are not sensitive to the position of CO perpendicularly to the surface. However, their use together with the <d_Pd–C_> (<d_Pd–C_>+CN and <d_Pd–C_>+GCN combinations) significantly enhanced the quality of prediction, with the best performance of their combination <d_Pd–C_>+CN+GCN.

ADF for Pd–C–Pd combinations (ADF_Pd–C–Pd_) demonstrated a similar quality as <d_Pd–C_>+CN+GCN and Coulomb matrix, while ADF for Pd–C–O (ADF_Pd–C–O_, Figure S3, Supplementary Materials) was slightly better. At that time, ADF was still less effective than RDF as a descriptor, but their use together resulted in the best obtained precision (RDF+ADF_Pd–C–O_).

**Figure 4:**
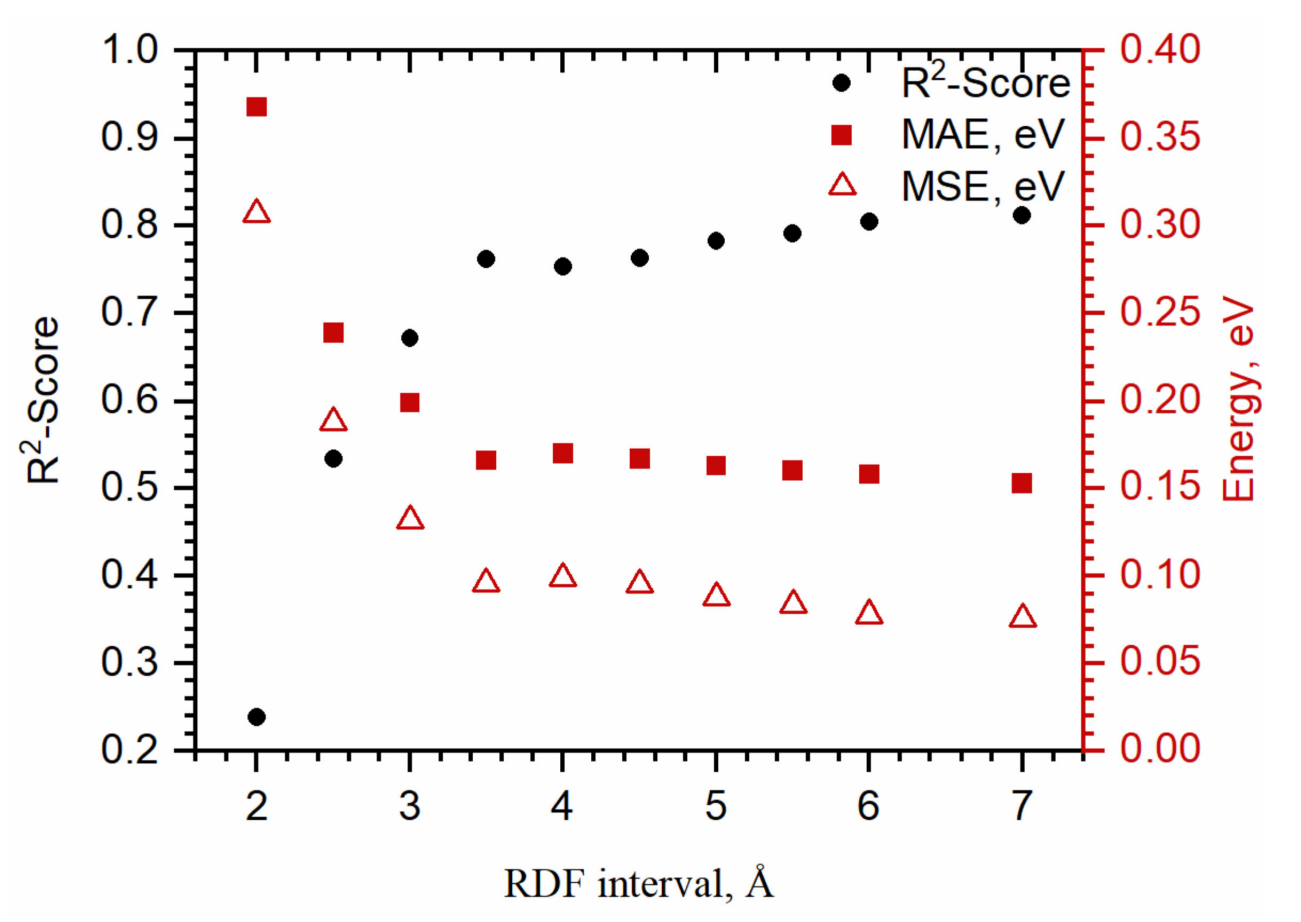
The quality of binding-energy prediction when using a different length of RDF.

We also tested which part of RDF contains the most valuable information about the structure (Section 4.2, Supplementary Materials). Reducing the interval from 0–7 to 0–3 Å had little effect on the prediction accuracy (Figure 4) but already on the interval 0.1–2.0 Å the quality decreased significantly. From 0 to 1.5 Å, the RDF have zero or near-zero values, and therefore are useless for training ML models.

We also split RDF into segments with a length of 1, 1.5, 2, 2.5, and 3 Å and tried them as descriptors (see Table S5, Supplementary Materials). The highest accuracy was provided by segments that include 1.5–2.5 Å interval, highlighting the impact of the local environment on the binding energy of the adsorbate and the principal applicability of the above approach to ultra-small particles without long-range order.

Extended parts of RDF cannot be used alone for precise energy estimation: for example, the interval from 4.0 to 7.0 Å leads to a more than twofold increase in MAE. However, their combined use together with the short-range intervals improves the quality of predictions and enables recovery of detailed information about the adsorption site.

### 3.3. Energy-Surface Construction

**Figure 5:**
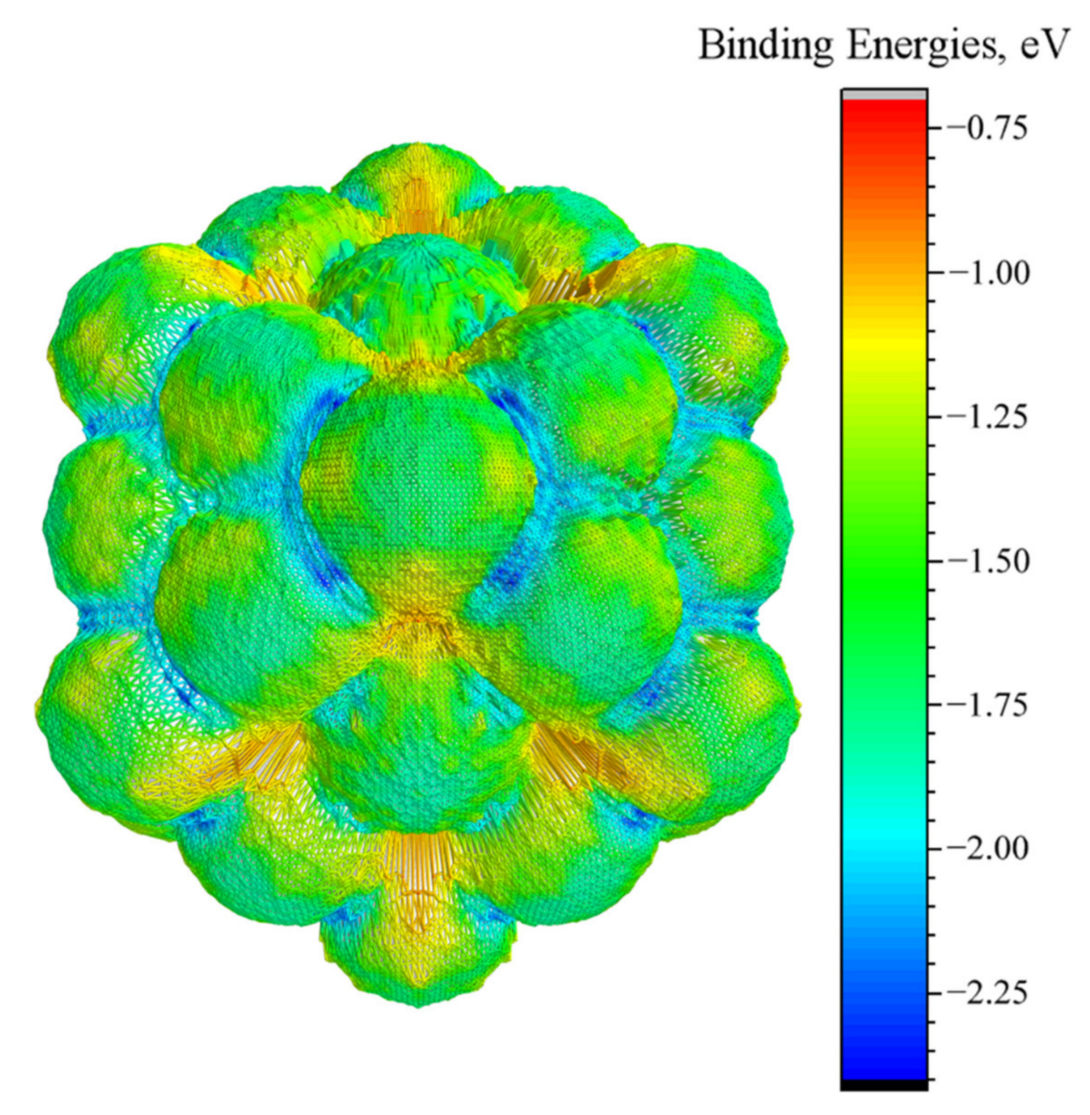
Energy surface of Pd_55_ predicted by trained SMV ML method. A grid with steps π/100 for φ and θ was used.

We applied the trained SVM model to predict the energy surface of the whole nanocluster. This surface illustrates the minimal values for the binding energy of the CO molecule adsorbed in each given point above the nanocluster. The energy surface estimated for Pd_55_ is shown in Figure 5.

According to these results, the lowest binding energies were found in the vicinity of three-fold hollow (111) and bridged sites on particle edges (from −2 eV to −2.41 eV, blue in Figure 5). Adsorption energy was slightly weaker near the bridged sites on (100) and (111) regular facets (from −1.8 eV to −2 eV, cyan), and top sites (from −1.25 eV to −1.9 eV, green). The highest energy was found for four-fold hollow sites at Pd (100) surface (ca. −0.8 eV, red).

These findings correlate with the results of ab initio DFT calculations (Figure 2a), where the CO molecule was strongly bound to the (111) hollow site (−2.64 eV in minimum) and bridged sites (from −2.40 eV for edge to −2.28 eV on (100) facet) and weakly bound to the top positions (ca. −1.8 eV). ML predicted higher energies than expected only in the case of three-fold hollow sites. This can be explained by the significant variation in the C–O distances in the probe molecule that were not taken into account in the training-set calculations.

Obtained data are also in good agreement with the results of the computational study [9] as well as with the experimentally observed shifts of absorption bands in FTIR spectra: the highest red shift is observed for CO on hollow sites and the lowest is for linearly adsorbed CO [54]. The same algorithm was also applied to construct the binding-energy surface for Pd_79_ and Pd_85_ clusters (Figure S6, Supplementary Materials).

## 4. Conclusions

In this work, we applied ML methods to the problem of adsorption-energy prediction. Among different ML methods, the SVM and ensemble methods (Extra trees, Gradient boosting) provided the best quality for prediction. RDF has shown its efficiency as a structural descriptor while mean Pd–C distances, CN, GCN, and ADF provided worse results. The space of structural parameters was probed by an adaptive sampling approach which ensured a good approximation quality for the regions with a strong variation in the target function. This approach enabled the prediction of the binding energy with the best precision using RDF as a descriptor: MAE of 0.15 eV (MSE 0.08 eV, R^2^-score 0.81) by the SVM method.

The further development of this methodology will permit quick and accurate prediction of binding energies along with the frequencies and intensities of atomic vibrations, which is crucial for quantitative analysis of infrared spectra and kinetic catalytic studies.

## Figures and Tables

**Table 1:** Comparison of the used ML algorithms in terms of their efficiency for predicting the binding energy over the entire interval of the calculated RDF.

ML Algorithm	MAE, eV	MSE, eV	R^2^-Score
Ridge regression	0.40	0.28	0.31
Decision tree	0.30	0.27	0.33
Lasso	0.39	0.26	0.36
AdaBoost	0.29	0.16	0.60
XGBoost	0.20	0.15	0.64
Gradient boosting	0.22	0.14	0.64
Random forest	0.22	0.14	0.65
Extra trees	0.19	0.13	0.68
SVM	0.15	0.08	0.81

## Data Availability

The data that support the findings of this study are available from the corresponding author, A.T., upon reasonable request.

## Supplementary Materials

The following supporting information can be downloaded online, Scheme S1. Algorithm of dataset point generation, Figure S1. Sites for CO adsorption on Pd55 cluster where potential scans were performed: (**a**) (100) top; (**b**) (100) bridge; (**c**) (111) bridge; (**d**) (111) hollow; (**e**) edge bridge; (**f**) vertex top. Pd atoms forming adsorbing site are outlined by the colored dashed line, Figure S2. Series of smeared RDF calculated for all structures of the ASTS, colored according to their binding energies, Figure S3. Series of smeared ADF calculated for all structures of the ASTS, colored according to their binding energies, Figure S4. MAE decrease upon adding new data points to the training sets prepared by different sampling approaches. The quality of predictions was estimated on the test set from randomly generated data points. Figure S5. Comparison of the quality of prediction of binding energy when using different descriptors and ML methods, Figure S6. Energy surface of (**a**) Pd79 and (**b**) Pd85 nanoclusters predicted by the SVM method trained on ASTS of Pd55. A grid with steps π/200 for φ and θ was used, Table S1. Positions of Pd atoms in Pd_55_ cluster, Table S2. Examples of data points in ASTS, Table S3. Comparison of the quality of prediction of binding energy when using different descriptors/ML methods, Table S4. Effect of varying the length of RDF range on the performance of the SVM method in binding-energy prediction, Table S5. Influence of the range of the RDF used for determination of binding energies by the SVM algorithm. References [55,56] are cited in the Supplementary Materials.

## References

[1] Pareek V., Bhargava A., Gupta R., Jain N., Panwar J. (2017). Synthesis and applications of noble metal nanoparticles: A review. Adv. Sci. Eng. Med..

[2] Cuenya B.R. (2010). Synthesis and catalytic properties of metal nanoparticles: Size, shape, support, composition, and oxidation state effects. Thin Solid Film..

[3] Vatti S.K., Ramaswamy K.K., Balasubramanaian V. (2017). Shape controlled palladium nano particles for hydrogenation of cinnamaldehyde. J. Adv. Nanomat..

[4] Sun C., Cao Z., Wang J., Lin L., Xie X. (2019). Shape and ligand effect of palladium nanocrystals on furan hydrogenation. New J. Chem..

[5] Takeguchi T., Manabe S., Kikuchi R., Eguchi K., Kanazawa T., Matsumoto S., Ueda W. (2005). Determination of dispersion of precious metals on CeO_2_-containing supports. Appl. Catal. A Gen..

[6] Shen M., Wei G., Yang H., Wang J., Wang X. (2013). Different selections of active sites for CO, C_3_H_6_, and C_10_H_22_ oxidation on Pd/CeO_2_ catalysts. Fuel.

[7] Lear T., Marshall R., Antonio Lopez-Sanchez J., Jackson S.D., Klapötke T.M., Bäumer M., Rupprechter G., Freund H.-J., Lennon D. (2005). The application of infrared spectroscopy to probe the surface morphology of alumina-supported palladium catalysts. J. Chem. Phys..

[8] Lamberti C., Zecchina A., Groppo E., Bordiga S. (2010). Probing the surfaces of heterogeneous catalysts by in situ IR spectroscopy. Chem. Soc. Rev..

[9] Yudanov I.V., Sahnoun R., Neyman K.M., Rösch N., Hoffmann J., Schauermann S., Johanek V., Unterhalt H., Rupprechter G., Libuda J. (2003). CO adsorption on Pd nanoparticles: Density functional and vibrational spectroscopy studies. J. Phys. Chem. B.

[10] Wang X., Wu G., Guan N., Li L. (2012). Supported Pd catalysts for solvent-free benzyl alcohol selective oxidation: Effects of calcination pretreatments and reconstruction of Pd sites. Appl. Catal. B Environ..

[11] Aleksandrov H.A., Neyman K.M., Hadjiivanov K.I., Vayssilov G.N. (2016). Can the state of platinum species be unambiguously determined by the stretching frequency of an adsorbed CO probe molecule?. Phys. Chem. Chem. Phys..

[12] Eichler A. (2003). CO adsorption on Ni (111)—A density functional theory study. Surf. Sci..

[13] Ouvrard A., Wang J., Ghalgaoui A., Nave S., Carrez S., Zheng W., Dubost H., Bourguignon B. (2014). CO Adsorption on Pd (100) Revisited by Sum Frequency Generation: Evidence for Two Adsorption Sites in the Compression Stage. J. Phys. Chem. C.

[14] Davis J.B., Horswell S.L., Piccolo L., Johnston R.L. (2015). Computational study of the adsorption of benzene and hydrogen on palladium–iridium nanoalloys. J. Organomet. Chem..

[15] Zeinalipour-Yazdi C.D., Willock D.J., Thomas L., Wilson K., Lee A.F. (2016). CO adsorption over Pd nanoparticles: A general framework for IR simulations on nanoparticles. Surf. Sci..

[16] Fan T.-E., Demiroglu I., Hussein H.A., Liu T.-D., Johnston R.L. (2017). DFT study of the structure, chemical ordering and molecular adsorption of Pd–Ir nanoalloys. Phys. Chem. Chem. Phys..

[17] Rankine C.D., Madkhali M.M., Penfold T.J. (2020). A deep neural network for the rapid prediction of X-ray absorption spectra. J. Phys. Chem. A.

[18] Timoshenko J., Lu D., Lin Y., Frenkel A.I. (2017). Supervised machine-learning-based determination of three-dimensional structure of metallic nanoparticles. J. Phys. Chem. Lett..

[19] Timoshenko J., Anspoks A., Cintins A., Kuzmin A., Purans J., Frenkel A.I. (2018). Neural network approach for characterizing structural transformations by X-ray absorption fine structure spectroscopy. Phys. Rev. Lett..

[20] Tupy S.A., Karim A.M., Bagia C., Deng W., Huang Y., Vlachos D.G., Chen J.G. (2012). Correlating ethylene glycol reforming activity with in situ EXAFS detection of Ni segregation in supported NiPt bimetallic catalysts. ACS Catal..

[21] Lansford J.L., Vlachos D.G. (2020). Infrared spectroscopy data-and physics-driven machine learning for characterizing surface microstructure of complex materials. Nat. Commun..

[22] Calle-Vallejo F., Martínez J.I., García-Lastra J.M., Sautet P., Loffreda D. (2014). Fast prediction of adsorption properties for platinum nanocatalysts with generalized coordination numbers. Angew. Chem. Int. Ed..

[23] Gasper R., Shi H., Ramasubramaniam A. (2017). Adsorption of CO on low-energy, low-symmetry Pt nanoparticles: Energy decomposition analysis and prediction via machine-learning models. J. Phys. Chem. C.

[24] Praveen C., Comas-Vives A. (2020). Design of an Accurate Machine Learning Algorithm to Predict the Binding Energies of Several Adsorbates on Multiple Sites of Metal Surfaces. ChemCatChem.

[25] Beachkofski B., Grandhi R. Improved distributed hypercube sampling. Proceedings of the 43rd AIAA/ASME/ASCE/AHS/ASC Structures, Structural Dynamics, and Materials Conference.

[26] Hruska E., Abella J.R., Nüske F., Kavraki L.E., Clementi C. (2018). Quantitative comparison of adaptive sampling methods for protein dynamics. J. Chem. Phys..

[27] Pérez A., Herrera-Nieto P., Doerr S., De Fabritiis G. (2020). AdaptiveBandit: A multi-armed bandit framework for adaptive sampling in molecular simulations. J. Chem. Theory Comput..

[28] Lookman T., Balachandran P.V., Xue D., Yuan R. (2019). Active learning in materials science with emphasis on adaptive sampling using uncertainties for targeted design. Npj Comput. Mater..

[29] Gastegger M., Behler J., Marquetand P. (2017). Machine learning molecular dynamics for the simulation of infrared spectra. Chem. Sci..

[30] Kresse G., Furthmüller J. (1996). Efficient iterative schemes for ab initio total-energy calculations using a plane-wave basis set. Phys. Rev. B.

[31] Kresse G., Furthmüller J. (1996). Efficiency of ab-initio total energy calculations for metals and semiconductors using a plane-wave basis set. Comput. Mater. Sci..

[32] Blöchl P.E. (1994). Projector augmented-wave method. Phys. Rev. B.

[33] Feibelman P.J., Hammer B., Nørskov J.K., Wagner F., Scheffler M., Stumpf R., Watwe R., Dumesic J. (2001). The CO/Pt (111) Puzzle. J. Phys. Chem. B.

[34] Mason S.E., Grinberg I., Rappe A.M. (2004). First-principles extrapolation method for accurate CO adsorption energies on metal surfaces. Phys. Rev. B.

[35] Mason S.E., Grinberg I., Rappe A.M. (2006). Adsorbate–Adsorbate Interactions and Chemisorption at Different Coverages Studied by Accurate ab initio Calculations: CO on Transition Metal Surfaces. J. Phys. Chem. B.

[36] Hammer B., Hansen L.B., Nørskov J.K. (1999). Improved adsorption energetics within density-functional theory using revised Perdew-Burke-Ernzerhof functionals. Phys. Rev. B.

[37] Perdew J.P., Burke K., Ernzerhof M. (1996). Generalized gradient approximation made simple. Phys. Rev. Lett..

[38] Perdew J., Burke K., Ernzerhof M. (1998). Perdew, burke, and ernzerhof reply. Phys. Rev. Lett..

[39] Papanikolaou K.G., Darby M.T., Stamatakis M. (2019). CO-induced aggregation and segregation of highly dilute alloys: A density functional theory study. J. Phys. Chem. C.

[40] Github.com. https://github.com/bjmorgan/vasppy.

[41] Jain A., Hautier G., Moore C.J., Ong S.P., Fischer C.C., Mueller T., Persson K.A., Ceder G. (2011). A high-throughput infrastructure for density functional theory calculations. Comput. Mater. Sci..

[42] Github.com. https://github.com/materialsproject/pymatgen.

[43] Muthukrishnan R., Rohini R. LASSO: A feature selection technique in predictive modeling for machine learning. Proceedings of the 2016 IEEE International Conference on Advances in Computer Applications (ICACA).

[44] Dietterich T.G., Kong E.B. (1995). Machine Learning Bias, Statistical Bias, and Statistical Variance of Decision Tree Algorithms.

[45] Cortes C., Vapnik V. (1995). Support-vector networks. Mach. Learn..

[46] Bennett K.P., Campbell C. (2000). Support vector machines: Hype or hallelujah?. ACM SIGKDD Explor. Newsl..

[47] Natekin A., Knoll A. (2013). Gradient boosting machines, a tutorial. Front. Neurorobotics.

[48] Geurts P., Ernst D., Wehenkel L. (2006). Extremely Randomized Trees. Mach. Learn..

[49] Chen T., Guestrin C. XGBoost: A Scalable Tree Boosting System. In Proceedings of the 22nd ACM SIGKDD International Conference on Knowledge Discovery and Data Mining (KDD’16).

[50] Freund Y., Schapire R.E. (1997). A decision-theoretic generalization of on-line learning and an application to boosting. J. Comput. Syst. Sci..

[51] Breiman L. (2001). Random forests. Mach. Learn..

[52] Pedregosa F., Varoquaux G., Gramfort A., Michel V., Thirion B., Grisel O., Blondel M., Prettenhofer P., Weiss R., Dubourg V. (2011). Scikit-learn: Machine learning in Python. J. Mach. Learn. Res..

[53] Scikit-learn Machine Learning in Python. https://scikit-learn.org/.

[54] Groppo E., Bertarione S., Rotunno F., Agostini G., Scarano D., Pellegrini R., Leofanti G., Zecchina A., Lamberti C. (2007). Role of the support in determining the vibrational properties of carbonyls formed on Pd supported on SiO_2_–Al_2_O_3_, Al_2_O_3_, and MgO. J. Phys. Chem. C.

[55] Virtanen P., Gommers R., Oliphant T.E., Haberland M., Reddy T., Cournapeau D., Burovski E., Peterson P., Weckesser W., Bright J. (2020). SciPy 1.0: Fundamental algorithms for scientific computing in Python. Nat. Methods.

[56] Github.com. https://github.com/nlesc-nano/auto-FOX.

