# Supplementary material for: Adsorption Sites on Pd Nanoparticles Unraveled by Machine-Learning Potential with Adaptive Sampling
Source: Molecules. 2022 Jan 6;27(2):357. doi: 10.3390/molecules27020357 (PMC8780420; doi:10.3390/molecules27020357)
Supplement: Supplementary file 1 [file molecules-27-00357-s001.zip › molecules-1521300-supplementary.pdf]

molecules

Supplementary Materials

Adsorption Sites on Pd Nanoparticles Unraveled by Machine-Learning Potential with Adaptive Sampling

Andrei Tereshchenko [1,*], Danil Pashkov [1,2], Alexander Guda [1], Sergey Guda [1,2], Yury Rusalev [1] and Alexander Soldatov [1]

[1] The Smart Materials Research Institute, Southern Federal University, 344090 Rostov-on-Don, Russia; pashkov@sfedu.ru (D.P.); guda@sfedu.ru (A.G.); gudasergey@gmail.com (S.G.); yuri.rusalev@gmail.com (Y.R.); soldatov@sfedu.ru (A.S.)

[2] Vorovich Institute of Mathematics, Mechanics, and Computer Sciences, Southern Federal University, 344058 Rostov-on-Don, Russia

* Correspondence: tereshch1@gmail.com

Table of Contents

1. Benchmark Structures for DFT Calculations 3

2. Details of Training and Test Set (ASTS and RSTS) Preparation 4

3. Examples of Data Points in ASTS 8

4. Dependence of the Quality of Binding Energy Prediction on Various Factors 13

4.1. Effectiveness of Adaptive Sampling compared with Random Sampling 13

4.2. Influence of Descriptors/ML Methods 14

4.3. Effect of RDF Range 17

5. Energy Surfaces Predicted for Pd_{79} and Pd_{85} Clusters 19

1. Benchmark Structures for DFT Calculations

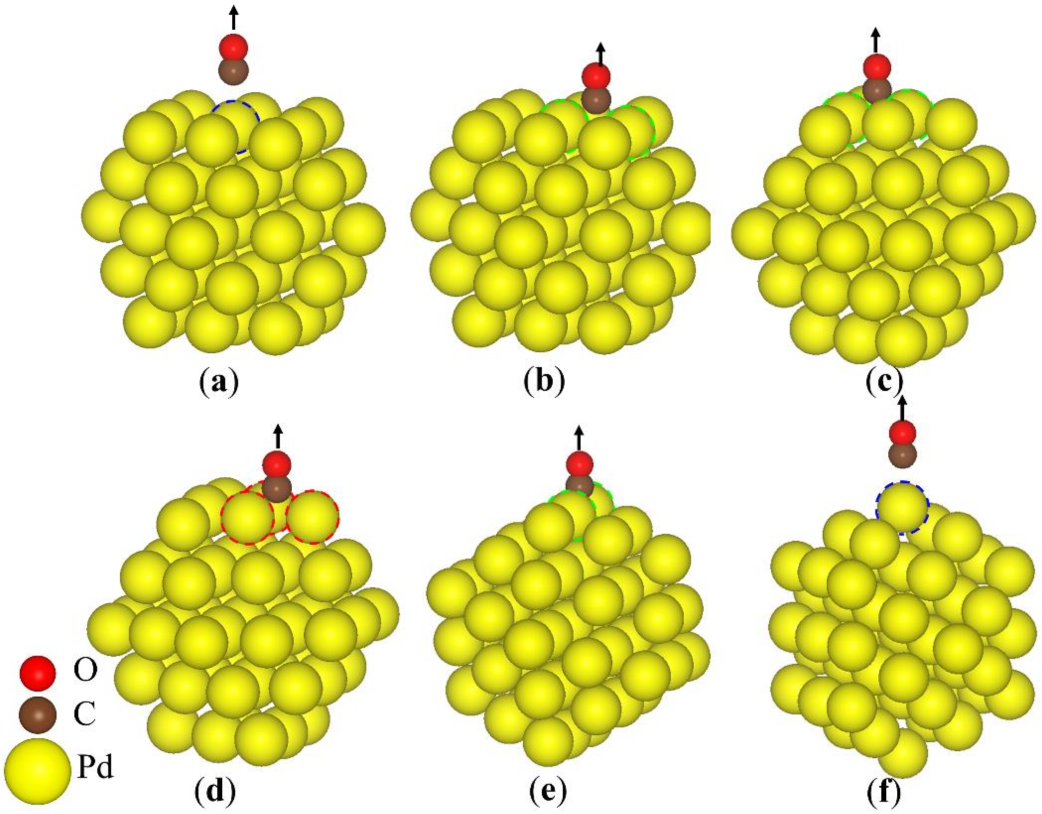

Figure S1. Sites for CO adsorption on Pd_{55} cluster where potential scans were performed: (**a**) (100) top; (**b**) (100) bridge; (**c**) (111) bridge; (**d**) (111) hollow; (**e**) edge bridge; (**f**) vertex top. Pd atoms forming adsorbing site are outlined by the colored dashed line.

2. Details of Training and Test Set (ASTS and RSTS) Preparation

To train ML methods it is necessary to collect a large number of features that describe objects under investigation and the responses corresponding to them. In this study, we focus on the use of radial distribution functions (RDF) and angular distribution functions (ADF) as descriptors of structure. All values of these functions were selected as features for training ML methods and binding energies were responses.

Positions of carbon atom in CO molecule near Pd NP were used as parameters of dataset structures (we will refer to them as dataset points or points), allowing reconstructing respective RDF or ADF and calculating binding energies. Spherical coordinates were used to describe these positions. As a result, the parameter space where the points for the training set were selected was presented by θ and φ angles, and the distance from the Pd NP surface to the carbon atom R_{Pd-C}. These parameters were generated in the following ranges: θ in [0, π], φ in [0, 2π] and R_{Pd-C} in [0, 1.4 Å] with a step π/100 for φ and θ. C–O distance ($\hat{R}_{c-o}$) was kept unchanged and equal to 1.128 Å.

To prevent the location of CO inside the Pd nanocluster, the movement of C was carried out from outside the nanocluster in direction to its center till specially introduced distance criteria d_{Pd-C} and d_{Pd-O} were not reached. The starting position was chosen at 12 Å (initial value of the parameter $\hat{R}$). Since the most distant Pd atoms in the Pd_{55} nanocluster were located at a distance of 6.71 Å from the center of the cluster, an additional 5 Å range above Pd surface safely includes all minimums of the potential energy surface for the CO/Pd system. Details of points generations for training set preparation are shown in Scheme S1.

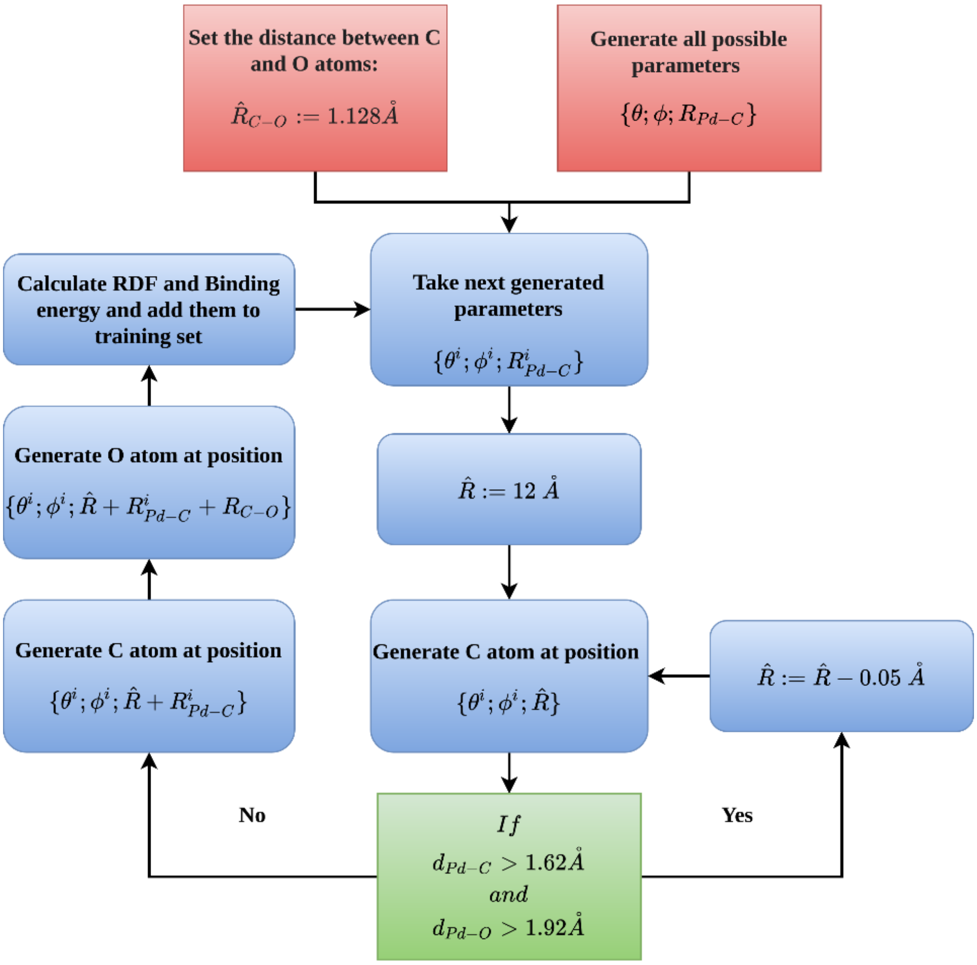

Scheme 1. Algorithm of dataset point generation.

For each data point in the training set (adaptive sampling training set, or ASTS) and test set (random sampling training set, RSTS), a set of descriptors was calculated, such as

mean Pd–C distances (<$d_{Pd–C}$>), coordination numbers (CN), generalized coordination numbers (GCN), RDF and ADF.

GCN of a surface site is estimated by considering not only the nearest neighbors of the adsorption site but also their coordination numbers as well by giving a weight normalized concerning the maximum coordination number. We used the approach described in [22] for their estimation.

RDF, or pair correlation function, describes how the particle density in a system varies as a function of distance from a reference particle (in our case, of Pd atoms relatively carbon atom). RDF were calculated using the vasppy [40] and pymatgen [41,42] packages for Python, in a radius from 0 to 7 Å with a step of 0.01 Å and convoluted using the Gaussian smearing implemented in scipy [55] package with default smearing parameter sigma of 10. All calculated RDF are shown in Figure S2.

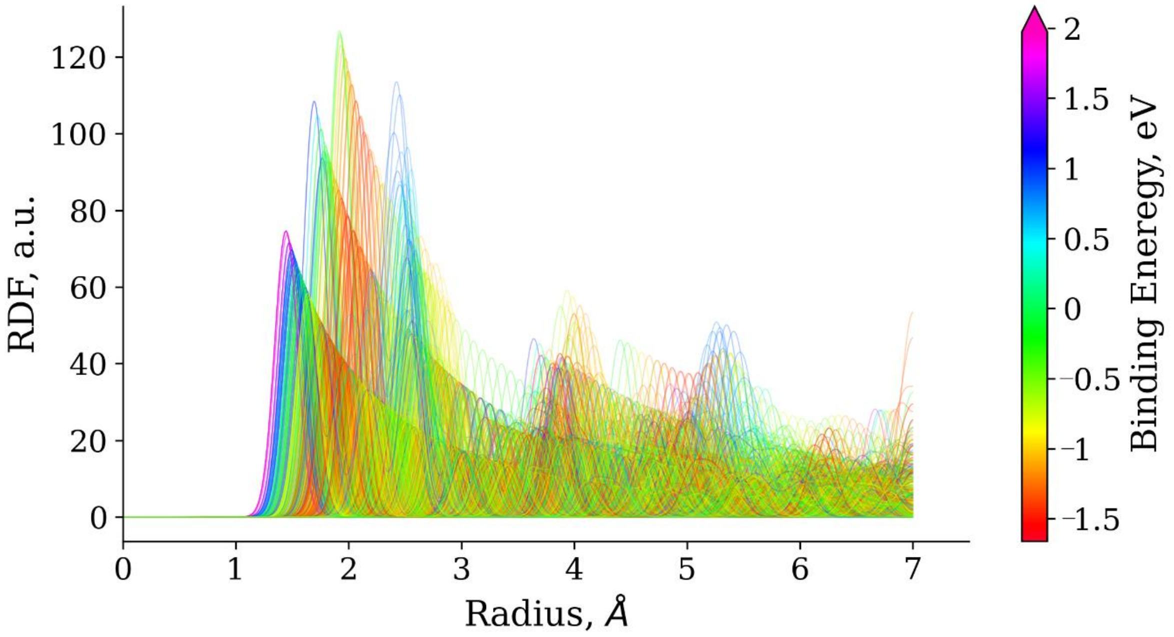

Figure S2. Series of smeared RDF calculated for all structures of the ASTS, colored according to their binding energies.

ADF is based on the fact that every combination of three atoms forms a triangle with three bond angles. To calculate ADF every combination of three atoms in a given system is iterated over with the determination of the bond angles, and those bond angles are added to a bin counter which represents some increment of 180 degrees of arc. After all bond angles are determined, each bin is divided by the total number of bond angles to obtain the probability that the bond angle between any three atoms will fall within that given increment of arc. ADF were obtained using the Auto-FOX package [56] for Python in the angular (phi) range from 0 to 180 ° with 1 ° increment. Angles were constructed for the maximum inter-atomic distance calculated individually for each structure as a maximum among the distances from carbon to the 5 nearest atoms of Pd. Obtained ADF were convoluted in the same way as RDF but with smearing parameter sigma of 5. The set of calculated ADF is shown in Figure S3.

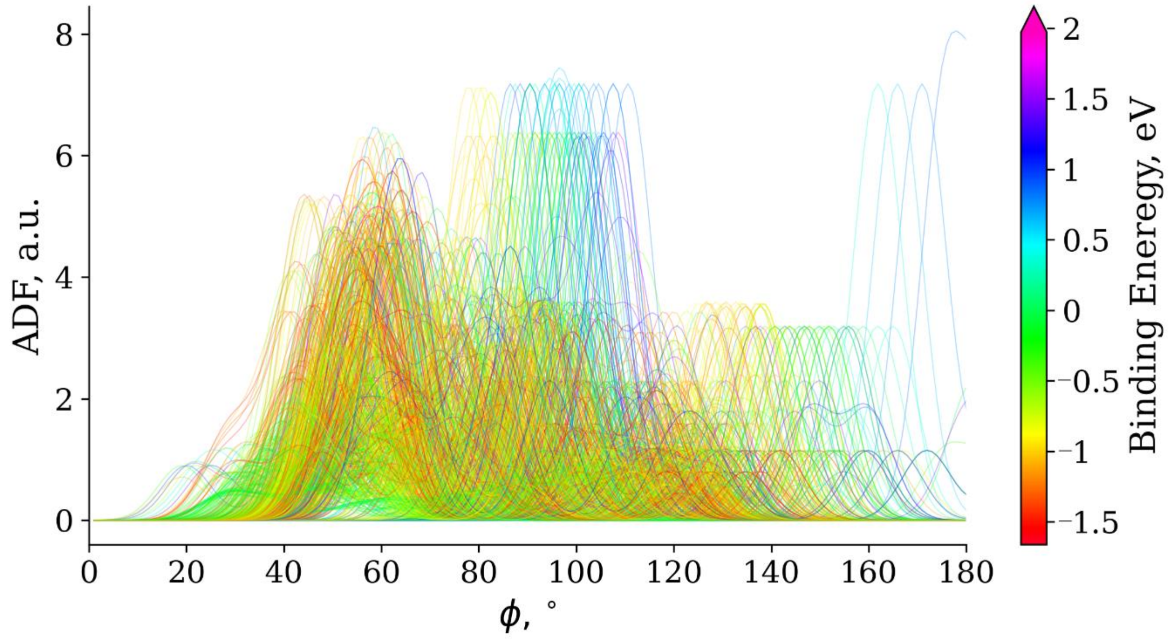

Figure S3. Series of smeared ADF calculated for all structures of the ASTS, colored according to their binding energies.

Structures for both ASTS and RSTS were prepared based on Pd_{55} nanocluster placed in 30 × 30 × 30 Å cell with fixed positions of Pd atoms (Table S1), near which CO molecules were generated following Scheme 1 the main text (for ASTS) or randomly in ranges θ in [0, π], φ in [0, 2π] and R_{Pd-C} in [0, 1.4 Å] (for RSTS).

Table S1. Positions of Pd atoms in Pd_{55} cluster.

№ of atom	x, Å	y, Å	z, Å
1	13.37280588	9.84801292	12.507595
2	20.15198637	16.62719342	12.507595
3	16.62719412	9.84801292	12.507595
4	9.84801363	16.62719342	12.507595
5	15	11.47520705	10.206395
6	15	11.47520705	19.793604
7	15	8.28283206	15
8	8.28283277	14.99999929	15
9	21.71716723	14.99999929	15
10	15	21.71716652	14.99999999
11	18.35858397	11.64141603	10.250245
12	18.35858397	11.64141603	19.74975399
13	11.64141674	18.35858326	10.250245
14	11.64141674	18.35858326	19.749754
15	11.64141603	11.64141603	10.250245
16	11.64141603	11.64141603	19.749754
17	18.35858326	18.35858326	10.250245
18	18.35858326	18.35858326	19.749754
19	13.37280588	9.84801291	17.492407
20	20.15198638	16.62719341	17.492407
21	16.73750985	13.26249015	12.54279
22	9.84801151	13.37280729	12.507595
23	16.627192	20.15198779	12.507595
24	16.62719412	9.84801292	17.492407

25	9.84801363	16.62719342	17.492407
26	20.15198849	13.37280729	12.507595
27	13.372808	20.15198779	12.507595
28	13.26249015	13.26249015	12.54279
29	15	11.52498029	15
30	18.52479437	15.0000014	10.206395
31	18.52479437	15.00000141	19.793604
32	11.47520562	15.00000141	10.20639501
33	11.47520563	15.00000141	19.793604
34	11.49534898	11.49534898	15
35	18.50465172	18.50465172	15
36	18.50465102	11.49534898	15
37	11.49534828	18.50465172	15
38	15	15	10.043675
39	15	15	19.95632599
40	9.84801151	13.37280729	17.492407
41	16.627192	20.15198779	17.492407
42	16.73750985	13.26249015	17.457211
43	13.26248944	16.73751056	12.54279001
44	13.26249015	13.26249015	17.457211
45	20.15198849	13.37280729	17.49240699
46	13.372808	20.15198779	17.492407
47	16.73751056	16.73751056	12.54279
48	11.52497959	15.00000071	15
49	18.47502041	15.00000071	15
50	14.99999999	18.52479579	10.206395
51	15	18.52479578	19.793604
52	15	15	15
53	13.26248944	16.73751057	17.4572110
54	16.73751056	16.73751057	17.457211
55	15	18.47502112	15

Examples of structures that make up ASTS with their descriptors and characteristics are shown in Section 3 of Supplementary Materials.

3. Examples of Data Points in ASTS

Here are presented some structures from ASTS with their description (Table S2). All data points were constructed following Scheme 1 of the main text.

Table S2. Examples of data points in ASTS.

<table>
<tr><td rowspan="11">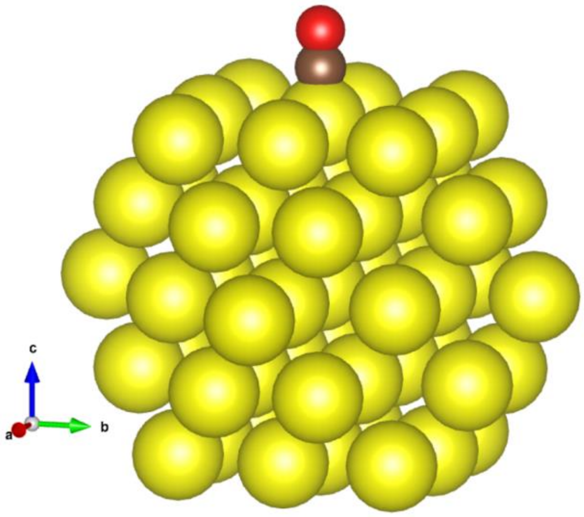
</td><td>№ in ASTS</td><td>000000</td></tr>
<tr><td>Description</td><td>atop adsorbed on central atom of Pd(100) facet</td></tr>
<tr><td>Cx, Å</td><td>15.000</td></tr>
<tr><td>Cy, Å</td><td>15.000</td></tr>
<tr><td>Cz, Å</td><td>21.576</td></tr>
<tr><td>Ox, Å</td><td>15.000</td></tr>
<tr><td>Oy, Å</td><td>15.000</td></tr>
<tr><td>Oz, Å</td><td>22.704</td></tr>
<tr><td>CN</td><td>1</td></tr>
<tr><td>GCN</td><td>6.33(3)</td></tr>
<tr><td><$d_{Pd–C}$> , Å</td><td>1.620</td></tr>
<tr><td></td><td>Binding energy, eV</td><td>−0.930</td></tr>
<tr><td>Structure</td><td colspan="2">Description</td></tr>
<tr><td>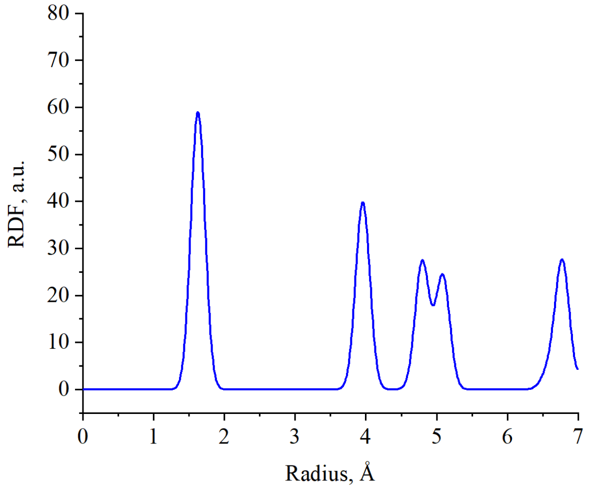
</td><td colspan="2">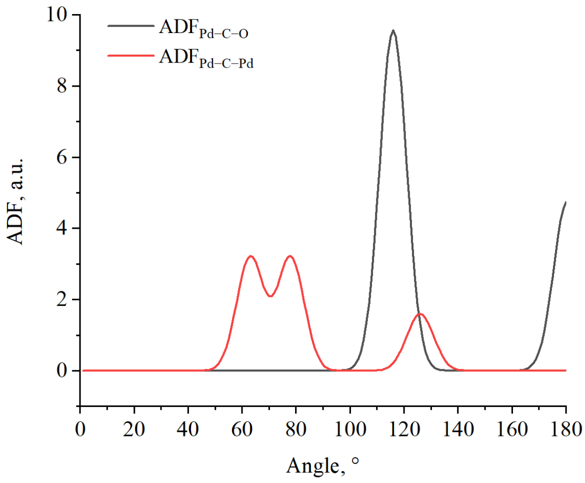
</td></tr>
<tr><td>RDF</td><td colspan="2">ADF</td></tr>
</table>

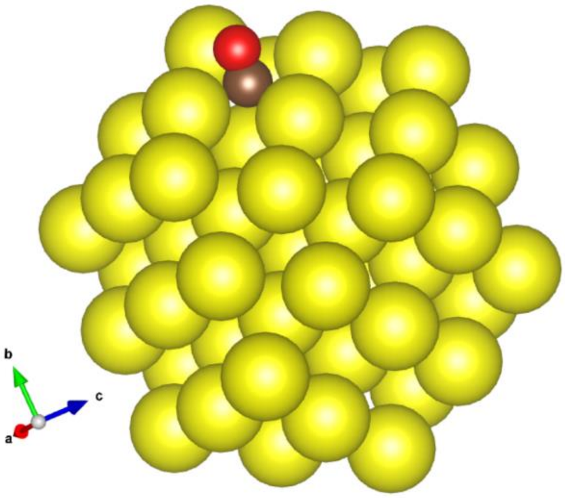

№ in ASTS	000002
Description	adsorbed on a 4-fold hollow site of Pd(100) facet
Cx, Å<br>Cy, Å<br>Cz, Å	13.120<br>20.223<br>14.738
Ox, Å<br>Oy, Å<br>Oz, Å	12.738<br>21.283<br>14.685
CN	4
GCN	7.231
$<d_{Pd\text{–}C}>$, Å	2.405
Binding energy, eV	−0.856

Structure	Description

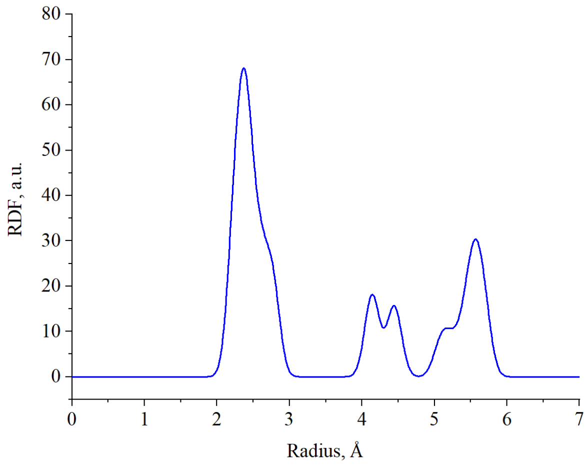

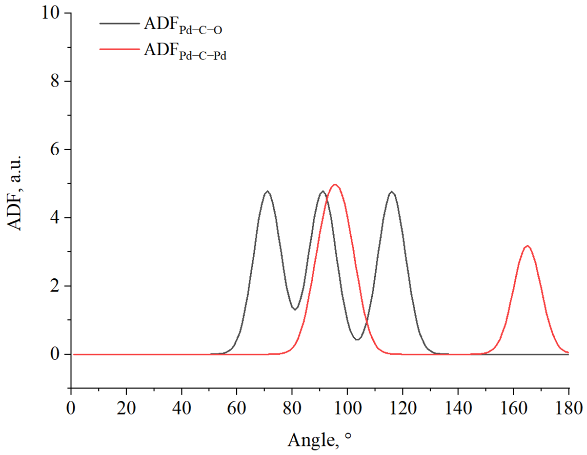

RDF	ADF

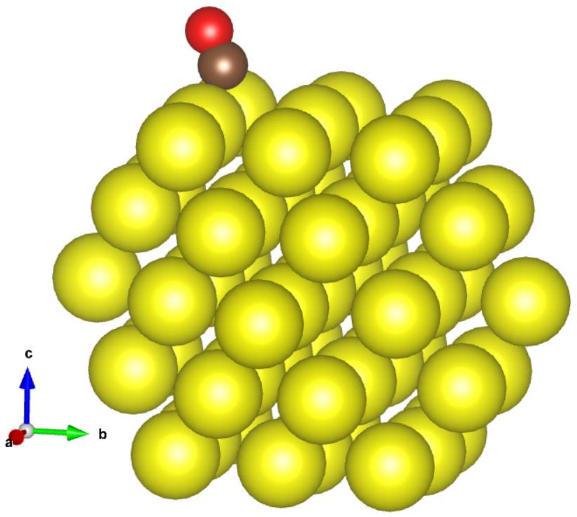

№ in ASTS	000004
Description	adsorbed between 2 atoms on edge Pd(100)/Pd(111) facets
Cx, Å	16.575
Cy, Å	11.360
Cz, Å	20.643
Ox, Å	16.833
Oy, Å	10.765
Oz, Å	21.566
CN	2
GCN	3.61(1)
$\langle d_{Pd-C} \rangle$, Å	1.904
Binding energy, eV	−1.767

**Structure**	**Description**

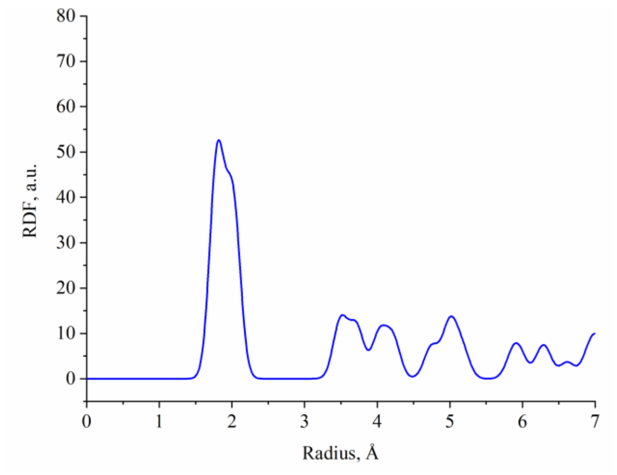

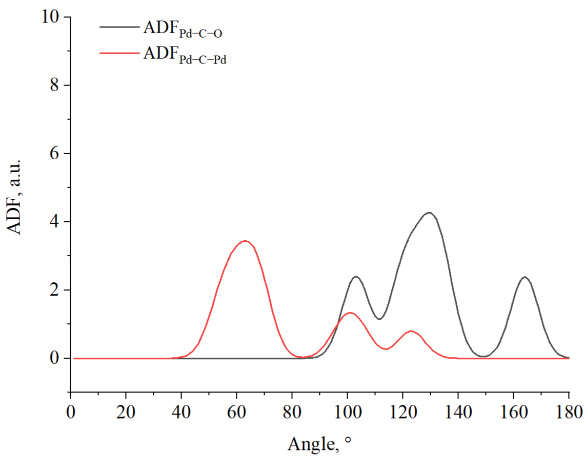

**RDF**	**ADF**

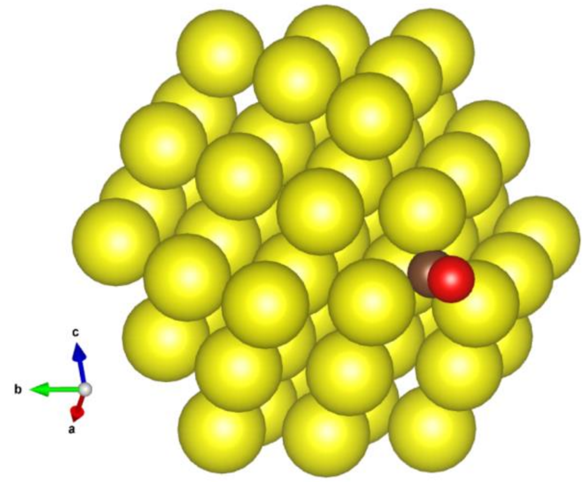

№ in ASTS	000019
Description	adsorbed on a 3-fold hollow site of Pd(111) facet
Cx, Å	10.713
Cy, Å	13.145
Cz, Å	10.750
Ox, Å	9.948
Oy, Å	12.814
Oz, Å	9.991
CN	3
GCN	3.773
$\langle d_{Pd-C} \rangle$, Å	1.962
Binding energy, eV	−1.130

Structure | **Description**

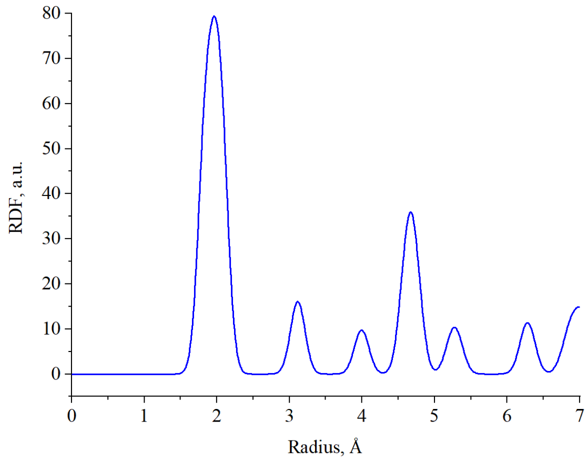

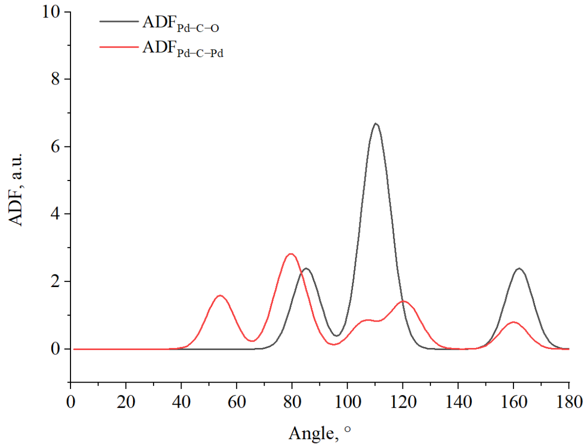

RDF | **ADF**

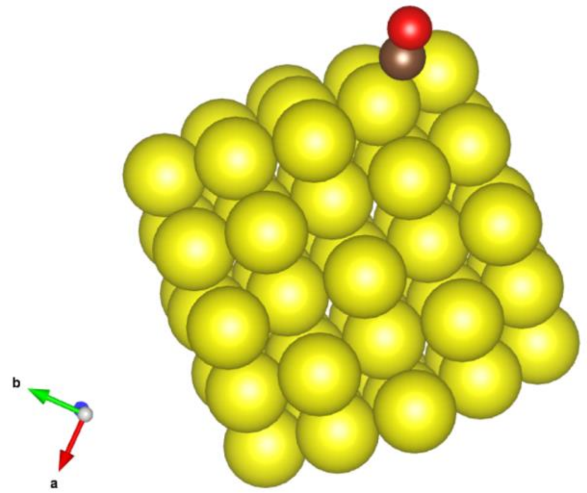

№ in ASTS	000547
Description	Atop adsorbed on edge Pd(100)/Pd(111) facets
Cx, Å<br>Cy, Å<br>Cz, Å	8.070<br>16.680<br>11.626
Ox, Å<br>Oy, Å<br>Oz, Å	7.079<br>16.920<br>11.143
CN	1
GCN	4.66(6)
$\langle d_{Pd-C} \rangle$, Å	1.985
Binding energy, eV	−1.485

**Structure**	**Description**

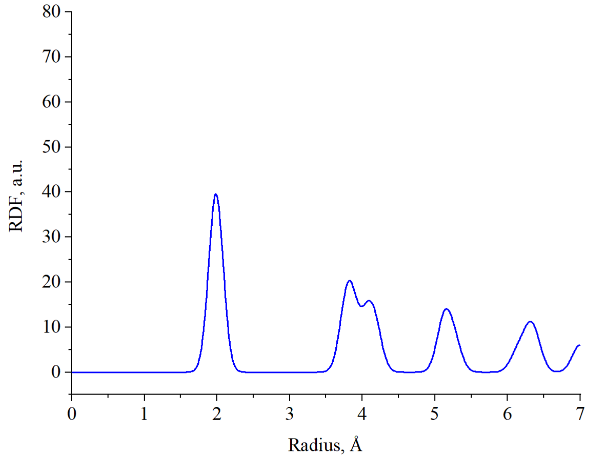

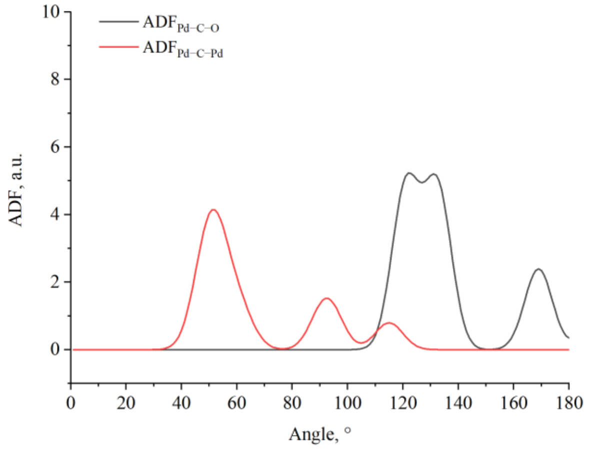

**RDF**	**ADF**

4. Dependence of the Quality of Binding Energy Prediction on Various Factors

Here we summarized the dependency of ML predictions quality on the size of the training set (ASTS and RSTS) used for ML (Figure S4), the use of different ML algorithms and descriptors (Table S3 and Figure S5), length and choice of different parts of RDF (Tables S4–S5).

4.1. Effectiveness of Adaptive Sampling compared with Random Sampling

To test the efficiency of the adaptive sampling procedure, we prepared an additional test set consisting of 250 data points, for the SVM ML algorithm trained preliminary on ASTS and RSTS data reported in the main text. This test set was prepared following the procedure used for RSTS. The comparison was performed upon increasing the size of training datasets (Figure S4).

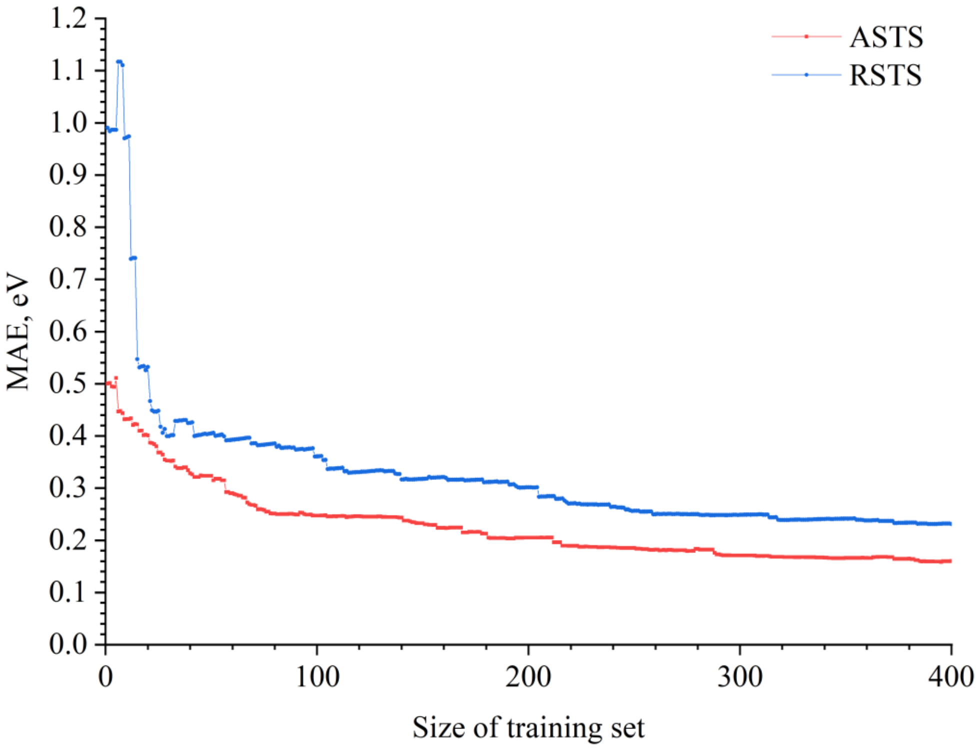

Figure S4. MAE decrease upon adding new data points to the training sets prepared by different sampling approaches. The quality of predictions was estimated on the test set from randomly generated data points.

As follows from Figure S4, ASTS demonstrates a lower mean absolute error of prediction requiring a smaller size of the training set.

4.2. Influence of Descriptors/ML Methods

Table S3. Comparison of the quality of prediction of binding energy when using different descriptors/ML methods.

Descriptor	ML Algorithm	MAE, eV	MSE, eV	R^2-score
	Ridge regression	0.44	0.38	0.05
	Decision tree	0.46	0.53	−0.32
	SVM	0.33	0.33	0.18
	Gradient boosting	0.36	0.33	0.18
$\langle d_{Pd–C} \rangle$	Extra trees	0.41	0.44	−0.09
	XGBoost	0.42	0.45	−0.12
	Random forest	0.41	0.41	−0.01
	**AdaBoost**	**0.38**	**0.30**	**0.27**
	Lasso	0.44	0.38	0.06
	Ridge regression	0.44	0.38	0.06
	Decision tree	0.33	0.38	0.05
	SVM	0.27	0.24	0.40
	**Gradient boosting**	**0.28**	**0.23**	**0.43**
$\langle d_{Pd–C} \rangle$+CN	Extra trees	0.31	0.33	0.17
	XGBoost	0.31	0.32	0.21
	Random forest	0.29	0.27	0.33
	AdaBoost	0.33	0.24	0.40
	Lasso	0.45	0.38	0.06
	Ridge regression	0.44	0.38	0.06
	Decision tree	0.38	0.43	−0.07
	SVM	0.35	0.33	0.19
	**Gradient boosting**	**0.31**	**0.27**	**0.33**
$\langle d_{Pd–C} \rangle$+GCN	Extra trees	0.31	0.30	0.26
	XGBoost	0.30	0.29	0.27
	Random forest	0.31	0.27	0.32
	AdaBoost	0.38	0.30	0.27
	Lasso	0.44	0.38	0.06
	Ridge regression	0.44	0.38	0.06
	Decision tree	0.33	0.39	0.03
	SVM	0.34	0.33	0.20
	**Gradient boosting**	**0.27**	**0.22**	**0.46**
$\langle d_{Pd–C} \rangle$+CN+GCN	Extra trees	0.28	0.27	0.33
	XGBoost	0.29	0.30	0.27
	Random forest	0.27	0.24	0.41
	AdaBoost	0.35	0.25	0.37
	Lasso	0.45	0.38	0.06
	Ridge regression	0.44	0.49	−0.20
	Decision tree	0.44	0.41	−0.02
	SVM	0.35	0.25	0.38
	Gradient boosting	0.36	0.24	0.40
Coulomb matrix	**Extra trees**	**0.32**	**0.21**	**0.48**
	XGBoost	0.34	0.24	0.41
	Random forest	0.34	0.23	0.43
	AdaBoost	0.45	0.32	0.21
	Lasso	0.43	0.43	−0.07
$ADF_{Pd–C–Pd}$	Ridge regression	0.43	0.31	0.23
	Decision tree	0.40	0.38	0.06

	SVM	0.30	0.20	0.51
	Gradient boosting	0.31	0.21	0.48
	**Extra trees**	**0.28**	**0.19**	**0.54**
	XGBoost	0.33	0.24	0.41
	Random forest	0.30	0.20	0.50
	AdaBoost	0.38	0.24	0.40
	Lasso	0.44	0.31	0.22
$ADF_{Pd–C–O}$	Ridge regression	0.40	0.28	0.31
	Decision tree	0.40	0.34	0.15
	SVM	0.28	0.17	0.58
	Gradient boosting	0.31	0.17	0.57
	Extra trees	0.26	0.15	0.64
	**XGBoost**	**0.27**	**0.15**	**0.64**
	Random forest	0.28	0.16	0.61
	AdaBoost	0.38	0.22	0.46
	Lasso	0.39	0.27	0.34
RDF	Ridge regression	0.40	0.28	0.31
	Decision tree	0.30	0.27	0.33
	**SVM**	**0.15**	**0.08**	**0.81**
	Gradient boosting	0.22	0.14	0.64
	Extra trees	0.19	0.13	0.68
	XGBoost	0.20	0.15	0.64
	Random forest	0.22	0.14	0.65
	AdaBoost	0.29	0.16	0.60
	Lasso	0.39	0.26	0.36
RDF+$ADF_{Pd–C–O}$	Ridge regression	0.26	0.16	0.60
	Decision tree	0.20	0.12	0.72
	SVM	0.15	0.07	0.82
	**Gradient boosting**	**0.15**	**0.06**	**0.86**
	Extra trees	0.15	0.06	0.85
	XGBoost	0.16	0.06	0.84
	Random forest	0.16	0.07	0.82
	AdaBoost	0.20	0.08	0.80
	Lasso	0.25	0.15	0.63

Data from Table S3 were partially illustrated in Figure 3 of the main text. Parts that were not included in the main text are shown in Figure S5 in comparison with results obtained using the SVM algorithm.

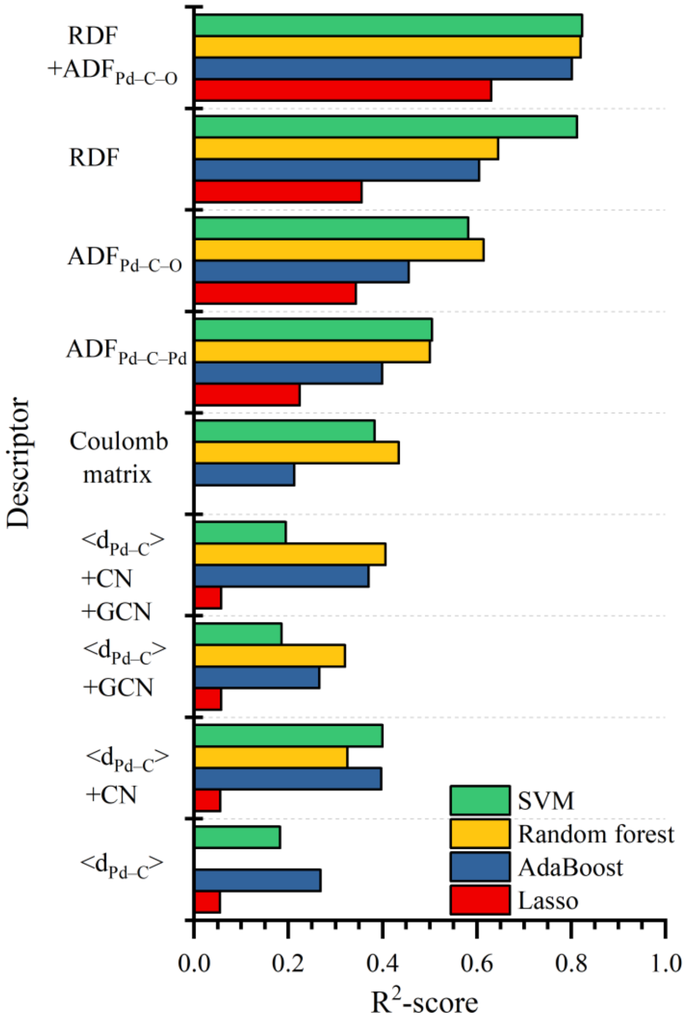

Figure S5. Comparison of the quality of prediction of binding energy when using different descriptors and ML methods.

4.3. Effect of RDF Range

Table S4. Effect of varying the length of RDF range on the performance of the SVM method in binding energy prediction.

Range, Å	MAE, eV	MSE, eV	R^2-score
**0–7.0**	**0.15**	**0.08**	**0.81**
0–6.0	0.16	0.08	0.81
0–5.5	0.16	0.08	0.79
0–5.0	0.16	0.09	0.78
0–4.5	0.17	0.10	0.76
0–4.0	0.17	0.10	0.75
0–3.5	0.17	0.10	0.76
0–3.0	0.20	0.13	0.67
0–2.5	0.24	0.19	0.53
0–2.0	0.37	0.31	0.24

Data from Table S4 were used in plot Figure 4 of the main text.

Table S5. Influence of the range of the RDF used for determination of binding energies by the SVM algorithm.

Length, Å	Range, Å	MAE, eV	MSE, eV	R^2-score
1	0.5–1.5	0.49	0.41	−0.01
	1.0–2.0	0.37	0.31	0.23
	**1.5–2.5**	**0.24**	**0.19**	**0.54**
	2.0–3.0	0.33	0.33	0.17
	2.5–3.5	0.45	0.40	0.01
	3.0–4.0	0.48	0.40	0.01
	3.5–4.5	0.52	0.48	−0.20
	4.0–5.0	0.51	0.45	−0.11
	4.5–5.5	0.44	0.36	0.12
	5.0–6.0	0.47	0.41	−0.01
	5.5–6.5	0.46	0.38	0.05
	6.0–7.0	0.48	0.40	0.02
1.5	0.5–2.0	0.37	0.31	0.24
	1.0–2.5	0.24	0.19	0.53
	**1.5–3.0**	**0.20**	**0.13**	**0.67**
	2.0–3.5	0.30	0.23	0.42
	2.5–4.0	0.42	0.36	0.12
	3.0–4.5	0.47	0.38	0.06
	3.5–5.0	0.47	0.38	0.06
	4.0–5.5	0.44	0.35	0.15
	4.5–6.0	0.42	0.31	0.22
	5.0–6.5	0.45	0.37	0.07
	5.5–7.0	0.44	0.36	0.11
2	0.5–2.5	0.24	0.19	0.53
	1.0–3.0	0.20	0.13	0.67
	**1.5–3.5**	**0.17**	**0.10**	**0.76**
	2.0–4.0	0.30	0.21	0.48
	2.5–4.5	0.42	0.33	0.18
	3.0–5.0	0.43	0.32	0.21
	3.5–5.5	0.42	0.32	0.21
	4.0–6.0	0.41	0.30	0.26
	4.5–6.5	0.39	0.29	0.28
	5.0–7.0	0.41	0.33	0.18
2.5	0.5–3.0	0.20	0.13	0.95
	1.0–3.5	0.17	0.10	0.96
	**1.5–4.0**	**0.17**	**0.10**	**0.96**
	2.0–4.5	0.27	0.17	0.74
	2.5–5.0	0.37	0.26	0.77
	3.0–5.5	0.39	0.27	0.78
	3.5–6.0	0.39	0.28	0.77
	4.0–6.5	0.38	0.27	0.69
	4.5–7.0	0.38	0.28	0.63
3	0.5–3.5	0.17	0.10	0.96
	**1.0–4.0**	0.17	0.10	**0.96**
	1.5–4.5	0.17	0.10	0.96
	2.0–5.0	0.27	0.16	0.78
	2.5–5.5	0.35	0.23	0.80
	3.0–6.0	0.37	0.25	0.81
	3.5–6.5	0.36	0.24	0.81
	4.0–7.0	0.37	0.27	0.71

5. Energy Surfaces Predicted for Pd_{79} and Pd_{85} Clusters

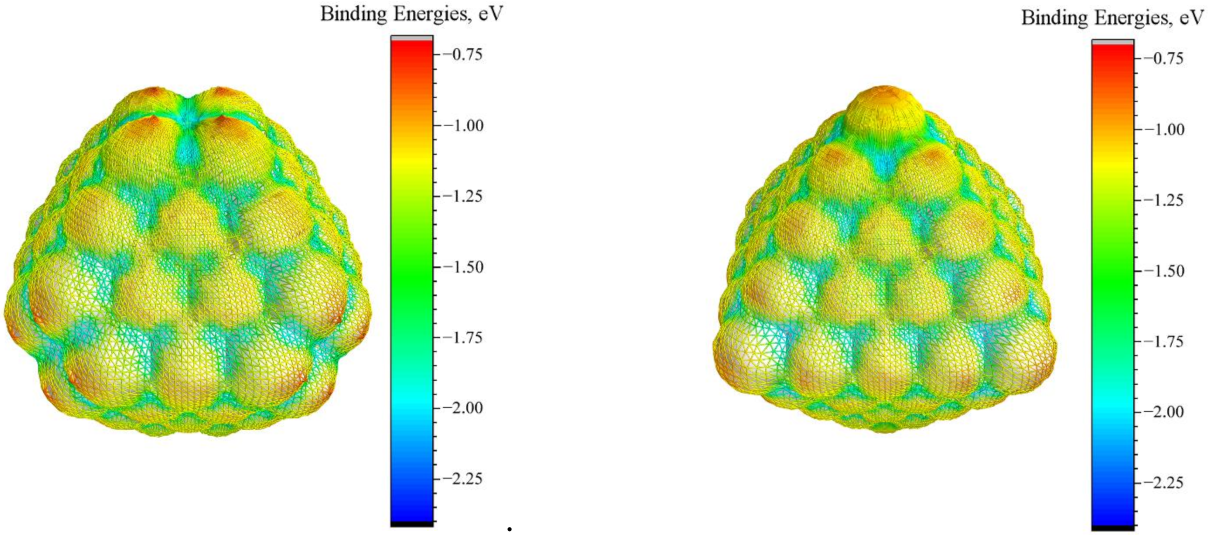

(**a**) (**b**)

Figure S6. Energy surface of (**a**) Pd_{79} and (**b**) Pd_{85} nanoclusters predicted by the SVM method trained on ASTS of Pd_{55}. A grid with steps $\pi/200$ for φ and θ was used.
